# From soil to cacao bean: Unravelling the pathways of cadmium translocation in a high Cd accumulating cultivar of *Theobroma cacao* L

**DOI:** 10.3389/fpls.2022.1055912

**Published:** 2022-12-02

**Authors:** Hester Blommaert, Anne-Marie Aucour, Matthias Wiggenhauser, Claudia Moens, Philippe Telouk, Sylvain Campillo, Jacques Beauchêne, Gautier Landrot, Denis Testemale, Serge Pin, Caleb Lewis, Pathmanathan Umaharan, Erik Smolders, Géraldine Sarret

**Affiliations:** ^1^ Université Grenoble Alpes, Université Savoie Mont Blanc, CNRS, Centre National de la Recherche Scientifique (CNRS), Institut de Recherche pour le Développement (IRD), Université G. Eiffel, Institut des Sciences de la Terre (ISTERRE), Grenoble, France; ^2^ Université de Lyon, Université Lyon 1, Ecole Normale Supérieure (ENS) de Lyon, Centre National de la Recherche Scientifique (CNRS), Unité Mixte de Recherche (UMR) 5276 Laboratoire de Géologie de Lyon - Terre, Planète et Environment (LGL-TPE), F-6922, Villeurbanne, France; ^3^ Institute of Agricultural Sciences, Eidgenössische Technische Hochschule (ETH) Zurich, Lindau, Switzerland; ^4^ Division of Soil and Water Management, Department of Earth and Environmental Sciences, Katholieke Universiteit (KU) Leuven, Leuven, Belgium; ^5^ Centre de Coopération Internationale en Recherche Agronomique pour le Développement (CIRAD), Unité Mixte de Recherche (UMR) Ecologie des Forêts de Guyane (EcoFoG), AgroParisTech, Centre National de la Recherche Scientifique (CNRS), Institut National de Recherche Pour l'agriculture, l'alimentation et l'environnement (INRA), Université des Antilles, Université de Guyane, Kourou, France; ^6^ Synchrotron SOLEIL, L’Orme des Merisiers, Saint-Aubin, Gif-sur-Yvette, France; ^7^ Univ. Grenoble Alpes, Centre National de la Recherche Scientifique (CNRS), Institut National polytechnique de Grenoble (INP), Institut Néel, Grenoble, France; ^8^ Université Paris-Saclay, Commissariat à l'énergie atomique et aux énergies alternatives (CEA), Centre National de la Recherche Scientifique (CNRS), Nanosciences et Innovation pour les Matériaux, la Biomédecine et l'Energie (NIMBE), Gif-sur-Yvette, France; ^9^ Cocoa Research Centre, University of the West Indies, St. Augustine, Trinidad and Tobago

**Keywords:** cadmium, cacao, translocation, stable isotope fractionation, imaging, speciation, LA-ICP-MS, XANES

## Abstract

The research on strategies to reduce cadmium (Cd) accumulation in cacao beans is currently limited by a lack of understanding of the Cd transfer pathways within the cacao tree. Here, we elucidated the transfer of Cd from soil to the nib (seed) in a high Cd accumulating cacao cultivar. Here, we elucidated the transfer of Cd from soil to the nib (seed) in a high Cd accumulating cacao cultivar through Cd stable isotope fractionation, speciation (X-Ray Absorption Spectroscopy), and localization (Laser Ablation Inductively Coupled Plasma Mass Spectrometry). The plant Cd concentrations were 10-28 higher than the topsoil Cd concentrations and increased as placenta< nib< testa< pod husk< root< leaf< branch. The retention of Cd in the roots was low. Light Cd isotopes were retained in the roots whilst heavier Cd isotopes were transported to the shoots (Δ ^114/110^ Cd _shoot-root_ = 0.27 ± 0.02 ‰ (weighted average ± standard deviation)). Leaf Cd isotopes were heavier than Cd in the branches (Δ ^114/110^ Cd _IF3 leaves-branch_ = 0.18 ± 0.01 ‰), confirming typical trends observed in annual crops. Nibs and branches were statistically not distinguishable (Δ ^114/110^ Cd _nib-branch_ = −0.08‰ ± 0.06 ‰), contrary to the leaves and nibs (Δ ^114/110^ Cd _nib-IF3 leaves_ = -0.25‰ ± 0.05 ‰). These isotope fractionation patterns alluded to a more direct transfer from branches to nibs rather than from leaves to nibs. The largest fraction (57%) of total plant Cd was present in the branches where it was primarily bound to carboxyl-ligands (60-100%) and mainly localized in the phloem rays and phelloderm of the bark. Cadmium in the nibs was mainly bound to oxygen ligands (60-90%), with phytate as the most plausible ligand. The weight of evidence suggested that Cd was transferred like other nutrients from root to shoot and accumulated in the phloem rays and phelloderm of the branches to reduce the transfer to foliage. Finally, the data indicated that the main contribution of nib Cd was from the phloem tissues of the branch rather than from leaf remobilization. This study extended the limited knowledge on Cd accumulation in perennial, woody crops and revealed that the Cd pathways in cacao are markedly different than in annual crops.

## Introduction

Cadmium (Cd) is a potentially toxic trace metal that has no known biological function in humans. It is known as a hitchhiker element, using transporters for essential elements such as Zn, Fe, Mn and Ca at all steps of these nutrient pathways, from root uptake to grain or seed loading (Clemens and Ma, 2016). Consequently, Cd eventually ends up in the human food chain. To protect consumers, the European Commission and the Codex Alimentarius approved new regulations, which set the maximum allowed Cd concentration in chocolates and cacao powders between 0.10 and 0.80 mg Cd kg^−1^ (EU, 2014; Codex Alimentarius Commission, 2018). These regulations have fueled research worldwide to monitor and mitigate Cd accumulation in cacao. There are indications that in the short-term agronomic changes like soil amendments could offer effective and sustainable strategies for Cd mitigation in cacao (Vanderschueren et al., 2021). In the long-term, breeding low Cd cultivars likely provides the highest potential. However, the genetics and breeding research is currently limited by the lack of understanding of how Cd is loaded into the developing cacao fruit of the tree.

*Theobroma cacao* L. is a perennial, woody tree that displays cauliflorous flowering, i.e. flowers and pods develop on the trunk and thicker branches of the plant (Wood et al., 2001). Cacao is considered a moderate Cd accumulator, as the Cd uptake potential of the cacao tree can be conceived as rather high compared to other agricultural crops, and more similar to woody species used for phytoextraction such as willow and poplar (Vanderschueren et al., 2021). Nonetheless, large differences (factor 13) in cacao bean Cd concentrations were found between cultivars grown in field conditions with comparable available soil Cd (Lewis et al., 2018). Some cultivars express thus high or low Cd accumulating properties. Due to its different anatomy, the mechanism of Cd accumulation may differ from other, more studied crops, like hyperaccumulators and cereals. In rice, the model plant for cereals, the knowledge of the pathway of Cd from soil to grain is in a very advanced stage compared to cacao. Numerous studies have shown the importance of membrane transport and pathways (e. g. xylem versus phloem transport), in the control of Cd in cereals. In summary, Cd is first taken up by root metal transporters for nutrients (McLaughlin et al., 2021). The roots are a major compartment for Cd accumulation since in the roots Cd is being sequestered into vacuoles. The Cd that is still mobile in the root is loaded into the xylem and exported into the shoot, and more particularly to the transpiring organs like the leaves (Clemens and Ma, 2016). When the grains develop, they can accumulate Cd from potentially two sources: direct transfer from roots or remobilization from plant organs that store mineral nutrients during vegetative plant growth (Yan et al., 2018). It is estimated that in rice, 91-100% of Cd accumulated in the grain is delivered *via* the phloem (Tanaka et al., 2007). Hence, Cd is remobilized into the grains from senescing leaves or stems *via* the phloem and/or in its pathway from root to shoot, Cd is transferred from xylem to phloem, which occurs likely in the rice nodes (Tan et al., 2019). Storage forms of Cd (i.e. its chemical speciation) have also been proven to play an important role in the control of the mobility of Cd in rice (Wiggenhauser et al., 2021a). Such knowledge of translocation pathways and storage forms of Cd within cacao would be an important step forward in understanding Cd accumulation in cacao beans.

In cacao, the overall pathway from soil to nib remains unclear. Moore et al. showed that Natural Resistance Associated Macrophage Protein 5 (NRAMP5) transporters may constitute an important pathway for uptake of Cd by cacao seedlings (Moore et al., 2020). They further suggested that the HMA-family proteins may contribute to Cd sequestration in root vacuoles, as shown in rice. A study on partitioning of Cd in cacao trees indicated that the transfer of Cd from root to the shoot tissues is large (Engbersen et al., 2019) compared to other crops where Cd accumulates mostly in the roots (Clemens et al., 2013; Vanderschueren et al., 2021). Based on concentration data they further proposed that Cd can be directly loaded from stems and branches into cacao beans without passing through leaves. This was corroborated by a study in mature cacao trees where the isotope fractionation suggested that Cd remobilization from the leaves is not an important pathway for Cd in the nibs, as has been shown for Cd loading into the rice grains (Barraza et al., 2019). However, the mechanism of Cd translocation in cacao trees remains unclear since organs such as roots and branches that may be important organs for storage and translocation were not analyzed. Little data on chemical Cd species has yet been obtained in cacao tissues. Synchrotron X-ray absorption spectroscopy (XAS) revealed that Cd in the nibs is coordinated to O, but S binding also occurred (Vanderschueren et al., 2020). However, information on Cd speciation, especially in important organs for storage and translocation is lacking.

The objectives of this study were to dissect potential mechanisms that control the accumulation and partitioning of Cd in a high Cd accumulator genotype of cacao grown in field conditions at environmentally relevant soil Cd concentrations. We determined the main compartments of Cd accumulation at the scale of the whole plant, using stable isotope and mass balance approaches. For the first time, the Cd isotope composition was measured in the roots, branches, and testa of a cacao tree. The corresponding fractionations helped in identifying plant uptake and translocation mechanisms. Speciation analyses in the branches, testa, and nibs and microscopic imaging of branches and roots provided complementary snapshots of major forms of Cd. In addition, interelement correlations were studied to elucidate nutrient pathways in the cacao tree. Finally, these data were combined to propose Cd transport pathways in cacao trees.

## Materials and methods

Sampling was conducted in January 2020 during the main harvest season, from November to January. Samples were collected at the International Cocoa Genebank, Trinidad (ICGT), a field cacao collection with approximately 2400 cacao genotypes, also further described as cultivars. A cultivar with high Cd accumulating properties, NA 312 of the genetic group Nanay (Motamayor et al., 2008), was selected for this study. The selection was based on the Cd levels in beans and leaves as reported in a previous study at the ICGT (Lewis et al., 2018). Three biological replicates, i.e. genetically identical trees, generated by rooted cuttings (T2, T4 and T6), were sampled.

### Location

The International Cocoa Genebank, Trinidad (ICGT) is situated in Centeno (Location using UTM – 685617E, 1,169,849N). The mean annual rainfall at this site is 2000 mm, and the mean annual maximum and minimum temperatures are 33°C and 22°C. The dry season takes place from January to May and the wet season from June to December. The soil in the ICGT is bordered by the Caroni river and is subject to repeated wet season floodings, which periodically bring sediments over the whole plot. The clay-loam soil (subgroup: Aquic Hapludalfs) is formed from non-calcareous silty claystone and its alluvial derivatives (Ahmad, 2011).

### Soil and tree sampling

Around each tree, a composite soil sample of the surface soil (0-20 cm) and the subsoil (20-40 cm) was collected with an auger around the dripzone of the cacao tree (1-2 m from trunk) by mixing three soil cores. The soil samples were afterward homogenized and weighed prior to oven drying (65°C). Around the surface roots (top 0-10 cm), the soil was dug out with a shovel (1-2 m from trunk) on three locations. Here, roots of different sizes were collected (with a diameter of 0.5-10 mm) (**Figure S1**). Branches, containing leaves of different maturation stages, were collected with pruning shears. The leaves were stripped off the branch and aliquots of branch tissue with a diameter of 3-7 mm were selected for further analyses (**Figure S1**). The leaves were categorized according to their maturation into Interflush-2 (IF2) and Interflush-3 (IF3) leaves. Interflush-2 leaves are leaves that have attained the deep green of a mature leaf, and have a green stem bearing the leaves. The maturation stage of Interflush-3 leaves begins when the stem bearing the leaves turns brown (Greathouse et al., 1971). About ten leaves of each age were collected (**Figure S1**). The cacao fruit or pod is made up of a large woody outer pod husk, filled with 20–50 cacao beans (seeds) (**Figure S1**). The cacao bean consists of a nib and an outer shell, the testa. The nib is the only part of the cacao bean that is retained during chocolate processing. Healthy fruits were collected and because of the limited number of fruits in the trees, mature but also intermediate and immature pods were collected. All plant samples (roots, branches, leaves, pods) were rinsed using baths with deionized water, and finally dried with a paper towel. First bulk root measurements indicated a contamination from soil particles (as seen by the high Mn and Fe content). To avoid contamination of soil particles in further analyses of the roots, the roots were peeled with a scalpel and remeasured. We assumed that the removed root part represented the epidermis and a minor part of the root cortex. Considering the minor biomass fraction that has been removed, the peeling did not affect the bulk concentration and isotope composition measurements in the roots. The intact pod husk was opened and all the different tissues [pod husk, placenta, testa, nib (**Figure S1**)] were separated and weighed. Immediately after separation, all plant samples were divided into subsets, apropos the further processing of the samples for the different techniques. For the X-ray absorption spectroscopy (XAS) analysis, an aliquot of the fresh tissues was milled with a pestle and a mortar in liquid N_2_ and stored in cryotubes in a freezer at -80°C until speciation analysis took place to preserve Cd speciation. Frozen-hydrated samples were shipped in a dry shipper filled with liquid N_2_. Aliquots for the concentration and isotope analyses were weighed for fresh weight/dry weight determination, homogenized, oven dried (65°C), shipped, and ground to powder with a grinder (Pulverisette7, Fritsch).

### Chemical and physical analyses

#### Soil and plant properties

Soil pH was measured in 0.01 M CaCl_2_, 1:5 soil to liquid (w:v). For organic carbon (expressed in %OC), the soil was acidified with 20-50 μL of 10% hydrochloric acid (HCl) and carbon was determined with elemental analyzer CarloErbaEA1108. The soil effective cation exchange capacity (eCEC) was determined using cobalt hexamine trichloride extractant solution as described in the standardized protocol ISO 23470:2018. Ammonium oxalate extractable aluminum, iron and manganese were measured in a 1:50 soil to oxalate (pH 3) extract ratio, where Al, Fe and Mn were analyzed in the extract by ICP-OES (Schwertmann, 1964). For the elemental content analyses, 50 mg of soil or plant were digested in respectively boiling aqua regia (2 mL 1:3 Suprapur HCl : HNO_3_) and concentrated nitric acid (2 mL Suprapur HNO_3_) in an open digestion block for 8 h at a maximum temperature of 130°C. The total elemental composition of the digest was measured by ICP-MS (Agilent7700x, Agilent Technologies) and ICP-OES (iCAP 7400 series, Thermo Scientific). For quality assurance, elemental concentrations were also determined in the certified reference materials BCR-142R (light sandy soil), NIST 1573a (tomato leaves), and NIST 2384 (baking chocolate). The Cd recovery of these samples ranged from 89 to 91% indicating that the digestion processes were effective. A full overview including other elements is given in **Table S1**.

Soil pools considered as phytoavailable Cd were extracted from the topsoil and subsoil using 0.05 M Ca(NO_3_)_2_ (5 g soil: 50 mL Ca(NO_3_)_2_), the extracts were shaken for 16 h, centrifuged, and then filtered (Rotilabo KH55.1 0.45 µm, Carl Roth) (Gray, 1998). Extracts were digested in concentrated HNO_3_ and total element content was measured with ICP-MS prior to further purification for isotope analyses.

#### Cd isotope analyses in soil and cacao tissues

Plant (200 ± 20 mg) and soil (400 ± 20 mg) samples selected for isotope analyses were digested with a microwave digestion system (MARS 6, CEM, North Carolina, USA) with respectively 8 mL concentrated nitric acid and 8 mL aqua regia. The total digestion cycle, consisting of a heating, a holding and a cooling phase, took 70 minutes to complete with a 20 min holding phase at 180°C. The soils were subjected to an additional digestion step with 3 mL HF and 3 mL HNO_3_ on the hotplate to ensure complete dissolution of the metals. Prior to the isotope analysis, Cd was purified using an anion exchange chromatography procedure (Cloquet et al., 2005; Pallavicini et al., 2014) which was previously used for samples in contaminated environments (Wei et al., 2016; Wiggenhauser et al., 2021b). Briefly, matrix elements (e.g. Ca, Zn, Sn) were removed from the sample using different concentrations of HCl while Cd was eluted from the column using 0.0012M HCl. Because the samples in this study were of environmentally-relevant conditions, the matrix contained a high element-to-Cd ratio. Thus, to achieve a better separation, it was necessary to perform the column chemistry twice. Some samples were purified three times if significant traces of Zn and Fe were still detected in the double purified Cd eluate. The yield of the purification procedure for certified reference materials can be found back in **Table S2**. The procedural blank (*n*=10) was <1.07%. Cadmium isotope compositions were measured using a multiple collector ICP mass spectrometer (MC-ICP-MS, Neptune Plus, Thermo Scientific) using an Aridus desolvator for sample introduction. Each sample was measured at least three times if there was enough sample available. To correct for instrumental mass bias, ‘standard-sample bracketing’ was applied and isotope standards and samples were doped with silver (Cd : Ag ratio of 2:1). Frequent measurements of a certified isotope standard and soil and plant standards for interlaboratory comparison revealed robust precision and accuracy for Cd concentration and Cd isotope measurements (**Tables S1**, **S2**).

#### Phytate content of cacao nibs

The phytate content in the nibs was determined in triplicate with a phytic acid assay (McKie and McCleary, 2016). In the SI the procedure and principle are explained. For quality assurance, an internal reference material (KULeuven wheat flour) was analyzed in triplicate. The recovery ranged between 105 and 110%. The internal reference material contained 0.88 g phytate/(100 g DW) (based on the median measured concentration after 38 individual analyses of the material).

#### Speciation analyses

Frozen hydrated samples were milled in liquid N_2_ using a cryo-grinder (Pulverisette23, Fritsch) and then pressed into pellets in cryo conditions (diameter 6 mm, thickness 3-5 mm). Cadmium K-edge XAS measurements were conducted at SAMBA beamline (SOLEIL synchrotron) and BM30 beamline (ESRF synchrotron). The X-ray absorption Absorption Near Edge Structure part (XANES), was analyzed since the diluted nature of the samples did not allow to retrieve valuable spectra from the Extended-X-ray Absorption Fine Structure part (EXAFS). The monochromator was a Si220 crystal, and acquisition was done in fly scan (SAMBA) and in step-by-step (BM30) mode. All samples were recorded at 20 K using a He cryostat, in fluorescence mode with a 35-element germanium detector (SAMBA) or a 13-element germanium detector (BM30). A Cd foil was recorded simultaneously in transmission mode for the energy calibration of XANES spectra.

A previously recorded database of Cd reference spectra recorded at 15 to 20K was used, containing Cd-cell wall, Cd-glutathione, Cd-phytochelatin, Cd-hydrated, Cd-malate, Cd-histidine, Cd phosphate. The preparation and measurements of this dataset are described in Huguet et al., 2012; Huguet et al., 2015. As additional standards, Cd-metallothionein extracted from mussels (Bataille et al., 2005), Cd phytate (phytate/Cd molar ratio = 5), Cd-Ca-oxalate (Ca-oxalate/Cd molar ratio = 10 and 50) (McBride et al., 2017) were measured at BM30 and included in the database. The absorption spectra of the references can be found in the SI (**Figure S2**).

The raw XANES spectra were averaged, calibrated in energy and normalized, and treated by linear combination fits (LCFs) using a XAS software [Fastosh (Landrot, 2018)]. LCF was performed by fitting the normalized XANES spectra in regions between −20 and 80 eV using the database of Cd reference compounds. The R-factor (= ∑[µ_exp_ – µ_fit_] ^2^ /∑[µ_exp_]^2^) was used to assess the goodness of fit. A combination of two reference compounds was used since the R-factor of the fit with three-reference compounds was not significantly smaller compared to the R-factor with two-reference compounds LCFs. For some spectra, there was not a unique solution. Particularly for the noisier spectra, the LCF procedure provided fits of equivalent quality. The fits of one XANES spectrum were considered equivalent if the R-factor increased <10% compared to the best fit. For these spectra, several fits are shown.

#### Imaging

Branches of two biological replicates (T2 and T4) were oven dried (60° C) prior to cutting with a microtome blade and placed on a glass slide with Kapton tape. Elemental analyses of the branch sections were achieved by Laser Ablation Inductively Coupled Plasma Mass Spectrometry (LA-ICP-MS). An Analyte Excite^+^ excimer laser ablation system (Teledyne) equipped with a 2‐Volume HelEx II Cell was used. The ARIS (Aerosol Rapid Introduction System, Teledyne) was operated to connect the 2-volume cell with the 8900 Triple Quadrupole ICP-MS (Agilent). The sections were ablated using an excimer laser at 193 nm with a 50 Hz repetition rate, spot size 10 µm square laser beam, a fluence of 3.5 J cm^-2^, and a scan speed of 50 µm s^-1^. Cadmium and other elements (C, P, Ca, Mn, Ni, Cu, and Zn) were measured with He cell gas to reduce polyatomic interferences. The program HDIP 1.6 was used to build the maps of the different isotopes. The final editing of the images was done with Fiji ImageJ (version 1.53s). For further calculations, elements were normalized to ^13^C in each pixel to correct for differences in tissue hardness and water content within the sample (Becker et al., 2008).

### Calculations and statistics

Isotope ratios of ^114^Cd/^110^Cd in the soil and cacao samples were expressed relative to the ^114^Cd/^110^Cd isotope ratio of isotope reference material NIST3108 as δ;


$$\delta^{114/110}Cd(‰)=\left[\frac{{\left(\frac{{\;}^{114}Cd}{{\;}^{110}Cd}\right)}_{sample}}{{\left(\frac{{\;}^{114}Cd}{{\;}^{110}Cd}\right)}_{NIST3108}}-1\right]\;x\;1000$$

The weighted average Cd isotope ratio of Cd in the entire trees was calculated as a weighted arithmetic mean;


$$\delta^{114/110}Cd_{tree}=\frac{\sum_{i}m_{i}c_{i}\delta^{114/110}Cd_{i}}{\sum^{}m_{i}c_{i}}$$

Where m_i_ denotes the dry weight (g), c_i_ the Cd concentration (mg kg^-1^) and δ_i_ the corresponding isotope ratio of the different cacao tissues i (roots, stem, branch, leaves and fruits). For the dry biomass of roots, stem, branch and leaves, values were used estimated by (Nurafiza et al., 2017) from an average of 20 cacao trees (10 years old). In **Table S3** more information concerning the mass balance estimation can be found.

Apparent Cd isotope fractionation between two compartments A and B is expressed as ‘Δ’ by subtracting Cd isotope ratios from each other;


$$\Delta^{114/110}Cd_{A-B}=\delta^{114/110}Cd_{A}-\delta^{114/110}Cd_{B}$$

Transfer factors (TF) indicate to what extent a crop accumulates Cd in its edible parts and are calculated as the ratio of the Cd concentration in either the plant leaf or the edible part (mg (kg DW)^-1^) to the aqua regia-extractable Cd concentration of the soil (mg (kg DW)^-1^) as shown below for the nib.


$$TF_{nib}=\frac{Cd_{nib}}{Cd_{soil}}$$

Similarly, internal translocation factors (ITF) were calculated to indicate how Cd is partitioned inside the crop. They are calculated as the ratio of the Cd concentration in a tissue, over the Cd concentration in another tissue (mg (kg DW)^-1^), as shown in next example;


$$ITF_{nib-branch}=\frac{Cd_{nib}}{Cd_{branch}}$$

In the images, we compared the accumulation of Cd in the different zones of the branches. Certain regions of interest (ROIs) were defined. The average of the ^13^C corrected-counts of Cd in these ROIs were normalized to the average ^13^C corrected-counts Cd counts in the ROI of the wood (**Table 4**). For example, the relative intensity of Cd in the pith was expressed as: (Cd_pith_/^13^C_pith_)/(Cd_wood_/^13^C_wood_). Additionally, the relation between Ca, Mn, Zn, Cu, Ni, and P was verified in these ROIs by making a regression prediction of the ^13^C-corrected counts. The extent of correlation was determined by the R^2^ of the bivariate fit of Y= log (E/^13^C) and X = log (Cd/^13^C).

The SD (standard deviation) reported throughout the study, was calculated with the biological replicates, i.e. the genetically identical trees. One-way analysis of variance (ANOVA) was conducted to compare means. Posthoc Tukey HSD tests were used when ANOVA revealed significant differences in the comparison of means (level of significance: *p*<0.05). Simple linear regression was used to test the relationship between Cd concentrations and nutrients (level of significance: *p*<0.05). The data sets were first tested for equality of variances with the O’Brien and Brown-Forsythe test (**Table S4**). Additionally, the normal distribution of residuals was tested with the Shapiro-Wilk test (**Table S4**). If these prerequisites were not fulfilled, the data were log transformed prior to conducting an ANOVA, as was the case for the molar ratios of the nutrients versus Cd in the bulk and image analyses. Statistical analysis was conducted with JMP^®^ Pro version 14.0.0 (SAS Institute 2018). For the Cd isotope measurements, the 2SD (two times standard deviation) of replicate measurements on the same sample was additionally shown to visualize analytical uncertainty. For the LA-ICP-MS analyses, only two replicates (T2 and T4) were measured due to high cost of analyses.

## Results

### Characterization of the cacao-soil system

The soil was previously characterized as a clayey loam soil (Lewis et al., 2018). The pH measured at the plot was 4.1 ± 0.1 (mean ± standard deviation of replicates), soil organic carbon (SOC) content was 1.9 ± 0.4%, and the eCEC was 11.6 ± 0.12 cmol_c_/kg. The overall variation in soil properties between the three plots was low (**Table S5**). The aqua regia soluble (0.22 ± 0.05 mg kg^-1^) and total Cd (0.28 ± 0.05 mg kg^-1^) concentrations in the topsoil were at the lower end compared to soil Cd concentrations reported in cacao producing areas in Latin America and Caribbean. A meta-analysis of *n*=12 studies indicated concentrations ranging from 0.22 to 10.8 mg Cd kg^−1^ soil (either determined by aqua regia, or aqua regia + HF, or HNO_3_ + H_2_O_2_)) (Vanderschueren et al., 2021). However, due to the strongly acidic nature of the soil and the rather low SOC, a large fraction (29%) of the Cd was extracted by Ca(NO_3_)_2_ (0.08 ± 0.02 mg kg^-1^). The subsoil was lower in aqua regia soluble Cd (0.06 ± 0.01 mg kg^-1^) than the topsoil, a trend observed in many cacao plantations (Chavez et al., 2015; Arévalo-Gardini et al., 2017; Barraza et al., 2017; Gramlich et al., 2018).

Based on a comparison of proposed normal, low and deficient concentrations of nutrients in cacao leaves (van Vliet and Giller, 2017), the sampled cacao leaves had adequate levels of Ca (17.33 ± 1.78 g kg^-1^ DW), but were moderately deficient in P (1.17 ± 0.14 g kg^-1^ DW) and severely deficient in K (8.35 ± 2.09 g kg^-1^ DW) (**Table S6**). Phytate measurements revealed that 84% of total P in the nib was in the form of phytate-P (4.40 ± 1.00 g phytate-P kg^-1^, *n*=5) (**Table 1**). Similar concentrations of phytate have been reported in cacao nibs (1.89 – 4.53 g phytate-P kg^-1^) (Vanderschueren et al., 2023) and are in the range of phytate concentrations reported for different foodstuffs (Schlemmer et al., 2009).

**Table 1:** Phytate and relative elemental content (DW) in the nibs (*n*=5).

		mean	SD
Phytate*	g kg^-1^	15.60	3.50
P-phytate**	g kg^-1^	4.40	1.00
**Cd**	mg kg^-1^	2.07	0.38
P***	g kg^-1^	5.28	1.15
**P-phytate/P**	(g kg^-1^)/(g kg^-1^)	0.84	0.07
**phytate/Cd**	(mol kg^-1^)/(mol kg^-1^)	1300	140
**phytate/(Cd+Zn+Fe)**	(mol kg^-1^)/(mol kg^-1^)	6.2	1.9

*Myo- inositol 1,2,3,4,5,6 hexakisphosphate.

**Phosphorus originating from phytate.

***Bulk P concentration.

### Cadmium accumulation, partitioning and Cd isotope ratios in the cacao-soil system

All cacao organs were highly enriched in Cd relative to the soil, with tissue-soil transfer factors ranging 10-28 depending on tissue. The Cd concentrations in the organs were ranging from 2.05 (nib) to 6.12 (branch) mg Cd kg^-1^ (**Table 2**). Branches were highly enriched in Cd, followed by the roots and leaves, and the lowest concentrations were found in the fruit tissues (testa, pod husk and nib).

**Table 2:** Cd concentrations and isotope compositions (as δ^114/110^Cd) for the soil and different cacao plant organs.

		Cd (mg/kg)				δ^114/110^Cd (‰)			
		n	mean	SD	group	n	mean	SD	group
**Topsoil**	Total*	3	**0.28**	0.05	d	3	**-0.32**	0.05	bc
	Ca(NO_3_)_2_	3	**0.08**	0.02	d	3	**-0.17**	0.03	ab
**Subsoil**	Total**	1	**0.10**		cd				
	Ca(NO_3_)_2_	3	**0.03**	0.01	d	2	**-0.18**	0.03	ab
**Roots**		3	**3.21**	0.87	b	2	**-0.58**	0.02	d
**Branch**		3	**6.12**	0.44	a	3	**-0.33**	0.03	bc
**Pod husk**	average	5	**2.76**	0.94	b	5	**-0.26**	0.06	ab
	immature	1	3.23			1	-0.31		
	intermediate	1	3.19			1	-0.31		
	mature	3	2.46	1.20		3	-0.22	0.05	
**Testa**	average	4	**2.36**	0.43	bc	4	**-0.28**	0.06	abc
	immature								
	intermediate	1	2.23			1	-0.21		
	mature	3	2.46	1.20		3	-0.30	0.05	
**Nib**	average	5	**2.05**	0.42	bc	3	**-0.41**	0.10	c
	immature	1	2.61			1	-0.44		
	intermediate	1	2.41			1	-0.41		
	mature	3	1.75	0.08		3	-0.40	0.13	
**IF2 leaves**		3	**3.49**	1.12	b	3	**-0.15**	0.03	a
**IF3 leaves**		3	**3.09**	0.35	b	3	**-0.18**	0.02	ab

*Total digestion (aqua regia with subsequent HF digestion).

**Aqua regia digestion.

**^$^** Ca(NO3)2-extract: 0.05 M Ca(NO_3_)_2_ (5 g soil: 50 mL Ca(NO_3_)_2_).

The standard deviation (SD) is calculated based on the mean of the biological replicates (genetically identical trees). Letters denote different statistical groups with the Tukey HSD test (*p ≤* 0.05). Immature, intermediate, mature are samples with different maturation stages in the development of the fruit. Values in bold indicate the average Cd concentration and isotope composition of the cacao tissues.

The Cd mass fractions in the trees revealed that branches contained most Cd (0.57 ± 0.01 [= mass_Cd, branch_(mg)/mass_Cd,tree_(mg), (**Table S3** and **Figure S3**)]. The stem followed (0.21 ± 0.01), assuming a similar Cd concentration as in the branches. Subsequently were the roots (0.14 ± 0.01), leaves (0.09 ± 0.01), and lastly the cacao fruit (<0.01). The Cd mass balance within fruits was calculated based on the measured dry weights (**Table S3**). The partitioning of Cd in the fruit was highly variable between the biological replicates but most Cd was found in the pod husk (0.62 ± 0.15), followed by the nib (0.29 ± 0.13) and only a minor fraction of fruit Cd was in the placenta (0.03 ± 0.01) and testa (0.06 ± 0.02) (**Figure S4**).

**Figure 1:**
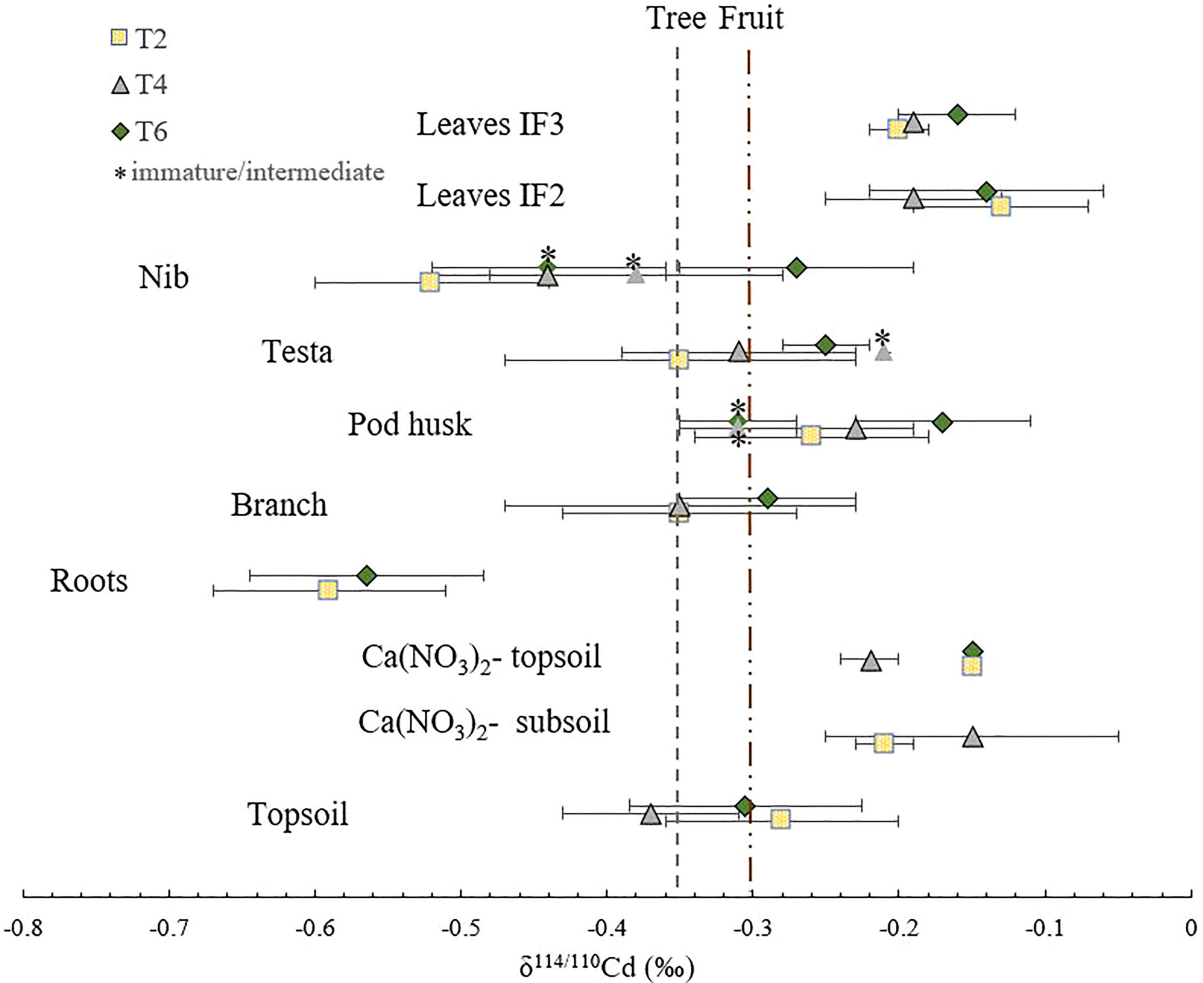
Cd isotope composition for the various organs in three trees (T2, T4 and T6). The first dashed line on the left represents the weighted average isotope composition of the whole cacao tree (δ^114/110^Cd = -0.35 ± 0.02 ‰ (*n*=2, biological SD)). The second dashed line represents the weighted average isotope composition of the cacao fruits (δ^114/110^Cd = -0.35 ± 0.05 ‰ (*n*=5, biological SD)). For the fruit tissues, the mature, intermediate and immature stages are indicated by depicting the immature and intermediate stages with a *. Error bars represent 2 x standard deviations (2 SD) of technical replicates (*n*=3, analytical precision). If no error bar is shown, no measurements of technical replicates were done.

The isotope compositions of total Cd in the topsoils of the three replicate trees were similar (δ^114/110^Cd _topsoil_ = -0.32 ± 0.05 ‰) (**Figure 1** and **Table 2**). A shift towards heavier isotopes was observed from the total topsoil Cd soil pool to the Ca(NO_3_)_2_-extractable pool (δ^114/110^Cd_extractable topsoil_ = -0.17 ± 0.03 ‰). The isotopic composition of the Ca(NO_3_)_2_-extractable pools of the subsoil and topsoil were similar (δ^114/110^Cd_extractable subsoil_ = -0.18 ± 0.03 ‰). The cadmium isotope composition varied widely within the plant. The average isotope composition of the whole cacao tree calculated based on biomass assumptions was δ^114/110^Cd = -0.35 ± 0.02 ‰ (*n*=2, weighted average). This composition was mostly governed by the isotope composition of the branches since they represent the biggest reservoir of Cd. No isotope fractionation was observed from total soil Cd to tree Cd (Δ^114/110^Cd_tree-topsoil_ = -0.05 ± 0.03 ‰). The trees were isotopically lighter than the Ca(NO_3_)_2_-available Cd source (Δ^114/110^Cd_tree-extractable soil_ = -0.20 ± 0.01 ‰).

Aerial tissues were isotopically heavier than roots, which is reflected in positive Δ^114/110^Cd _shoot-root_ = 0.27 ± 0.01 ‰ (**Table 3**). Within the shoot, Cd isotopes in the leaves were heavier than the branches (Δ^114/110^Cd _IF3 leaves-nib_ = 0.18 ± 0.01 ‰). No fractionation occurred between the branch and the fruit (Δ^114/110^Cd_fruit-branch_= 0.05 ± 0.02 ‰). The Cd in the cacao nibs was isotopically lighter than in the leaves (Δ^114/110^Cd _leaf IF3-nib_ = 0.25 ± 0.05 ‰). Within the fruit, Cd fractionated from the pod husk to the nibs (Δ^114/110^Cd_nib-pod husk_ = -0.19 ± 0.05 ‰). The testa showed an isotope composition in between the nibs and pod husks (Δ^114/110^Cd_testa-pod husk_ = -0.08 ± 0.01 ‰).

**Table 3:** Apparent Cd isotope fractionation Δ^114/110^ Cd _A-B_ in the cacao tree with SD of three biological replicates.

	n	mean	SD
tree-topsoil*	2	-0.05	0.03
tree-Ca(NO_3_)_2_ pool**^$^**	2	-0.20	0.01
shoot-root	2	0.27	0.01
fruit-branch	3	0.05	0.02
**branch-root**	**2**	**0.26**	**0.04**
pod husk-branch	3	0.07	0.05
testa-pod husk	3	-0.08	0.01
**nib-pod husk**	**3**	**-0.19**	**0.07**
nib-branch	3	-0.08	0.06
**IF3 leaves-branch**	**3**	**0.18**	**0.01**
**IF3 leaves-nib**	**3**	**0.25**	**0.05**
IF3 leaves-IF2 leaves	3	-0.03	0.03

*Total digestion (aqua regia with subsequent HF digestion).

**^$^** Ca(NO_3_)_2_-extract: 0.05 M Ca(NO_3_)_2_ (5 g soil: 50 mL Ca(NO_3_)_2_).

Fractionations that are significant according to Tukey HSD test (*p*<0.05) on the isotope composition tissues in **Table 2** are given in bold.

### Transfer of nutrients relative to Cd

The linear relationship between Cd and nutrients was evaluated using the log transform of bulk Cd and nutrient concentrations in the various organs (**Figure S5**). Zinc, Mn, Ca and Mg (hereafter referred to as Group I) correlated positively with Cd (**Table S7**). Contrasting trends were found for P, Cu, S and K (Group II) which correlated negatively to Cd.

**Figure 2:**
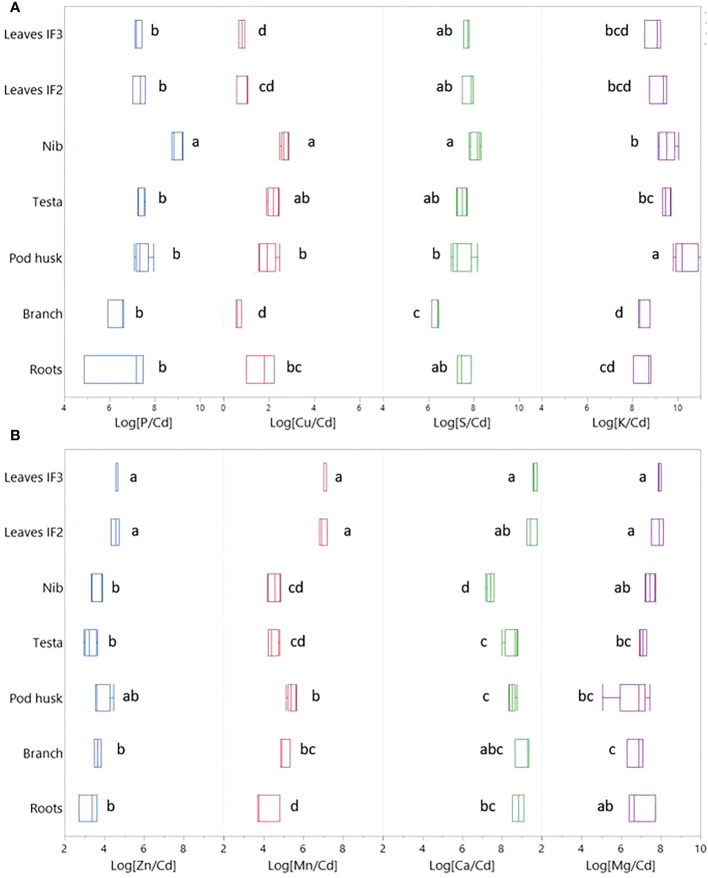
Bulk concentration ratios (log[nutrient/Cd (mg kg^-1^ DW^-1^/mg kg^-1^ DW^-1^)]) for the different tree organs. The value of each treatment is shown (*n*=3, except for fruit tissues where *n*=5). Letters denote statistical difference of the mean determined by Tukey HSD test (*p ≤* 0.05). **(A)** Group I elements that correlate positively with Cd. **(B)** Group II elements that correlate negatively with Cd (**Figure S3**).

For Group I, the log transformed ratios of nutrient versus Cd concentrations (**Figure 2**) in the different organs revealed that there was no clear retention of Cd relative to other nutrients in the roots. Likewise, in the branches, pod husks, testa and nibs, the element ratios had similar values, indicating a similar accumulation of Cd compared to the other elements. However, the nutrients of Group I accumulated markedly more in leaves than in the other tissues. For Group II, Cu and S were retained more in the roots than Cd, and accumulated less strongly in the branches compared to the nutrients in Group I. P/Cd ratios were markedly higher in the nibs than in the other tissues, whilst the K/Cd ratio was remarkably high in the pod husk.

### Cadmium speciation in cacao organs

**Figure 3:**
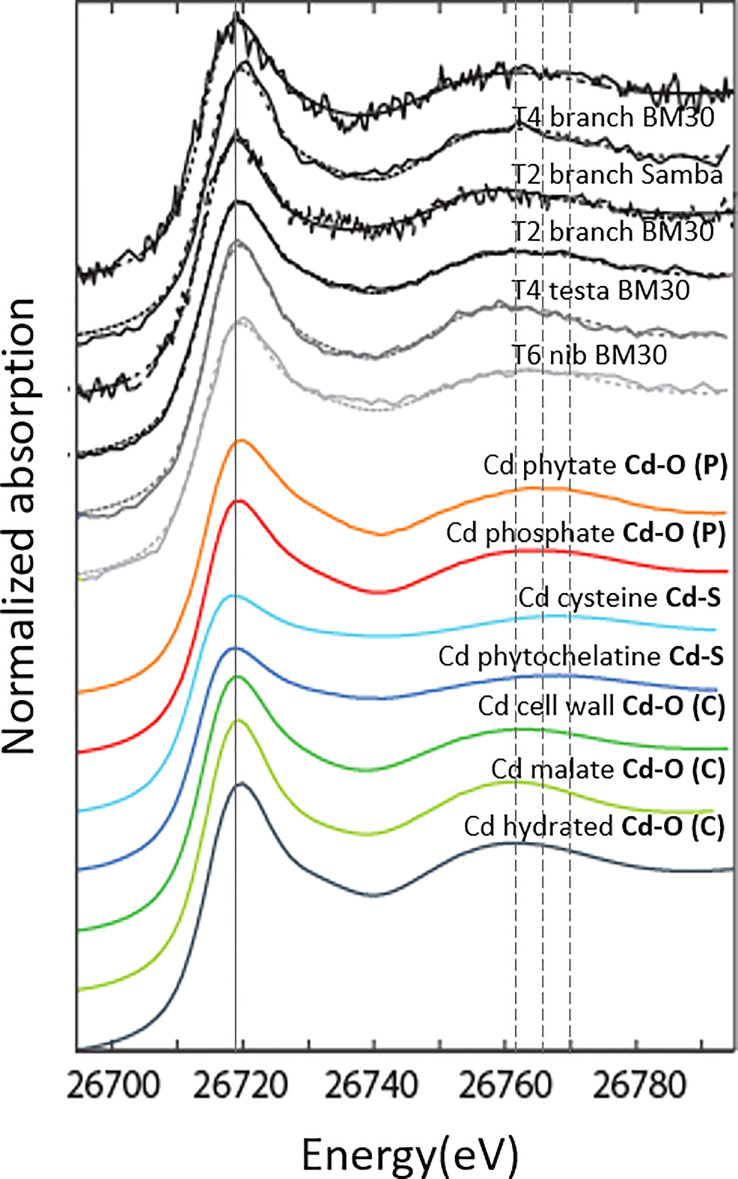
Cd K-edge XANES spectra of cacao tissues and of some Cd references. Cd-hydrated, Cd-malate, Cd-cell wall represent O (C)-ligands; Cd-phosphate and Cd-phytate represent O (P)-ligands, Cd-cysteine and Cd-phytochelatine represent S-ligands. The solid line is positioned at the white line energy (i.e. the intense absorption peak after the edge). The first vertical dashed line is at the first oscillation maximum for O (C)-ligands, the second dashed line for O (P)-ligands, and the third dashed line for S-ligands. Samples were either measured at ESRF, beamline BM30 or synchrotron Soleil, beamline SAMBA.

In the cacao tree system studied, we identified three major groups of ligands that bind Cd in cacao; (i) O (C)-ligands represented by Cd-hydrated, Cd-cellulose, Cd-cell wall, Cd-Ca-oxalate and Cd-organic acids mix, (ii) O (P)-ligands constituting of R-PO_4_H_2_ ligands represented by Cd-phosphate and Cd-phytate and (iii) S-ligands represented by Cd-phytochelatin, Cd-cysteine, Cd-glutathione and Cd-metallothionein. Groups (i) and (ii) represent O donors (Cd-O) and group (iii) S donors (Cd-S) in the first shell. For Cd-S, the amplitude of the white line (the intense absorption peak after the edge; **Figure 3**) is reduced compared to Cd-O. All spectra recorded resemble a first Cd coordination sphere constituted of O atoms (Cd-O). For these ligands, the second shell also contains valuable information as the oscillations for the O (P) and S-containing ligands shift to higher energy values than those for O (C)-ligands (**Figure 3**) as shown in Isaure et al., 2015. The branches and testa had similar energy of the first oscillation as the O (C)-ligands, while the first oscillation of the nib is shifted like the O (P)-ligands.

**Figure 4:**
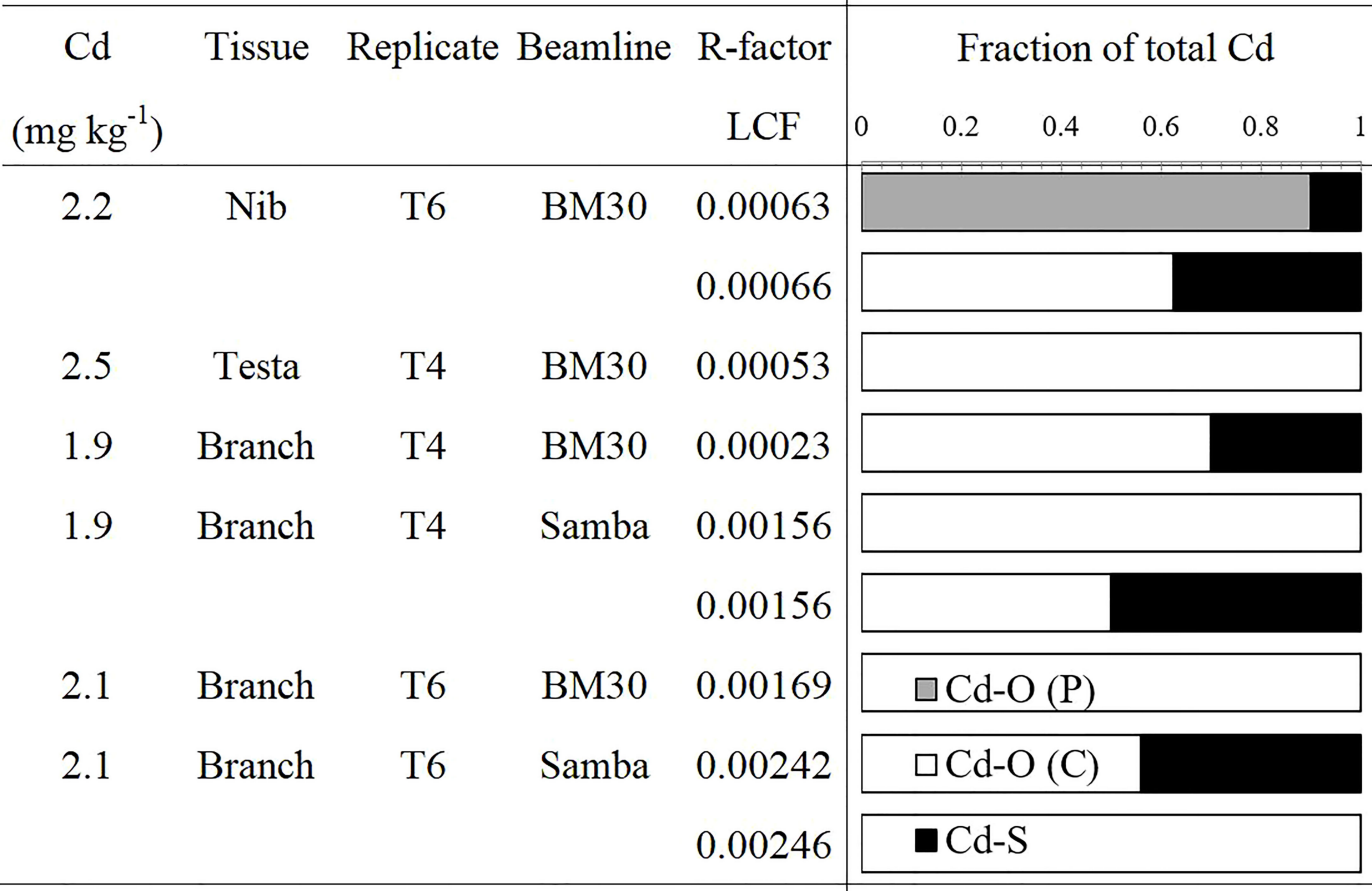
Proportion of Cd species in cacao tissues determined by XANES. Linear combination fits were done in the [E_0_-20; E_0_+80] eV range. Fit quality was examined by the R-factor. If the R-factor increases <10%, the fits are considered of equivalent quality and both results are shown in the figure.

Linear combination fits revealed that Cd in nib T6 was mostly (60 - 90%) bound to O-ligands (**Figure 4**), with S-ligands as minor species. The best fits were obtained with O (P)- ligands as major species, but also an equivalent fit was obtained with O (C)-ligands as major species (increase in R-factor of 4%). In testa T4, Cd was exclusively associated with O (C)- ligands (100%). The branches T4 and T6 were recorded both at BM30 and SAMBA beamlines. The measurements at BM30 showed that the Cd speciation in the biological replicates varied (T4: 60% versus T6: 100% O (C)-ligands). The spectra obtained at SAMBA were noisier (higher R-factor) but resulted in equivalent fits for the same measurement, between 60 and 100% O (C)-ligands and S-ligands as minor ligands. Hence, XANES revealed that overall, Cd was mostly bound to O -ligands in the branches, testa and nibs.

### Cadmium localization in branches

**Figure 5:**
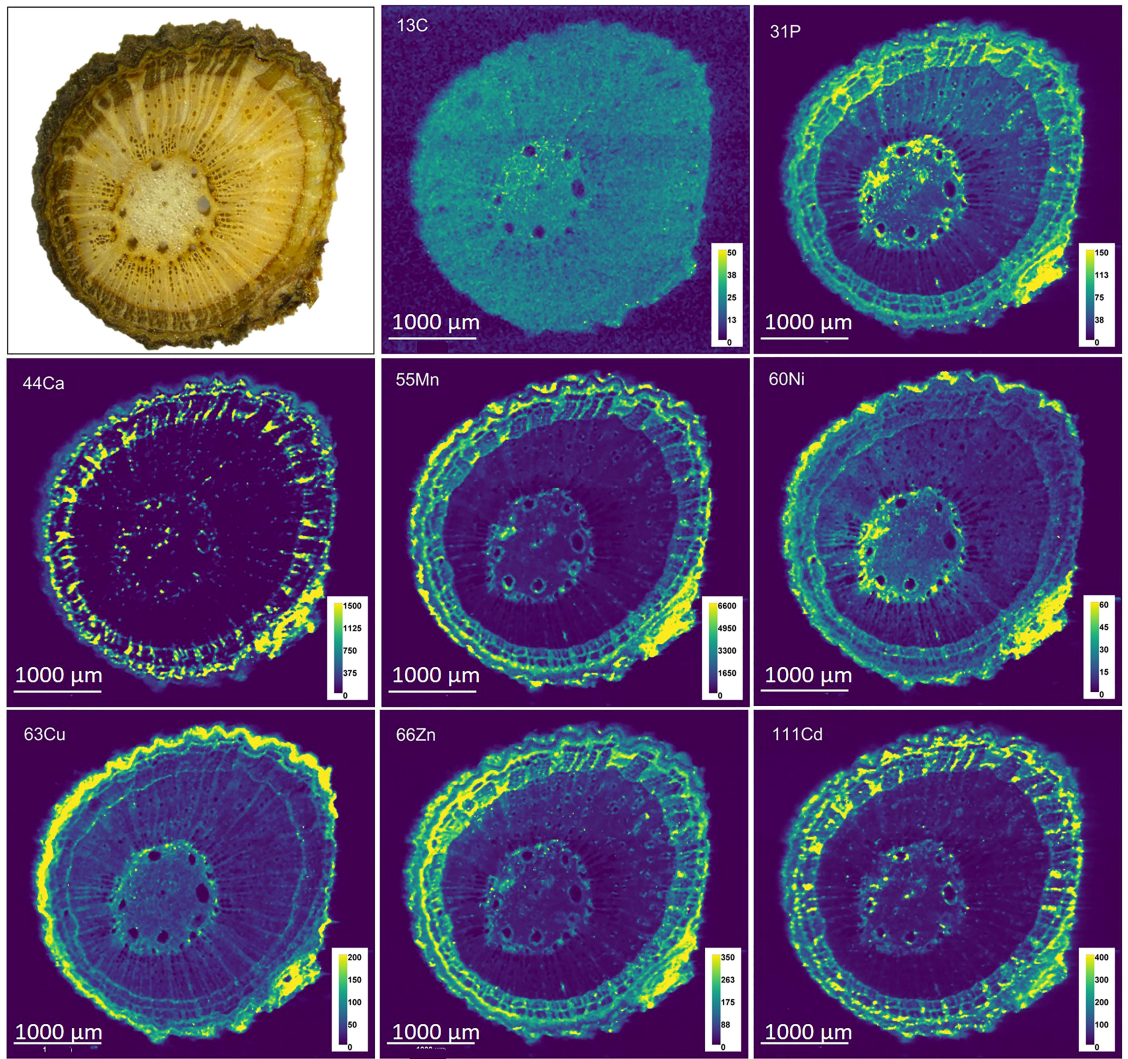
Microscope image and LA-ICP-MS elemental maps of cross section of branch T2. Cd, Ca, Mn and Zn co-localized, and less spatial correlation was found between Cd and P or Cu. The intensity is expressed in total counts (cts). The color scale is adjusted to the range of each element.

Branch cross sections were analyzed for Cd, Zn, Cu, Mn, Ca, Ni and P with LA-ICP-MS. The elemental maps revealed the relative abundances of an element between each part of the branch (**Figure 5**; **Figure S7**). In the branches of T2 and T4, the pith (medulla), wood and bark phloem, separated by the cambium could be identified (**Figure S6**). The wood contains xylem vessels which are in close contact with ray cells. These xylem rays are abundant and continue into the phloem and are essential for the translocation of nutrients between phloem and xylem. Phloem rays separate the phloem vessels, which consist out of sieve tubes, companion cells, phloem fibers and phloem parenchyma. The bark is surrounded by the periderm including phelloderm, cork cambium and heavily suberized cork (Brooks and Guard, 1952).

**Figure 6:**
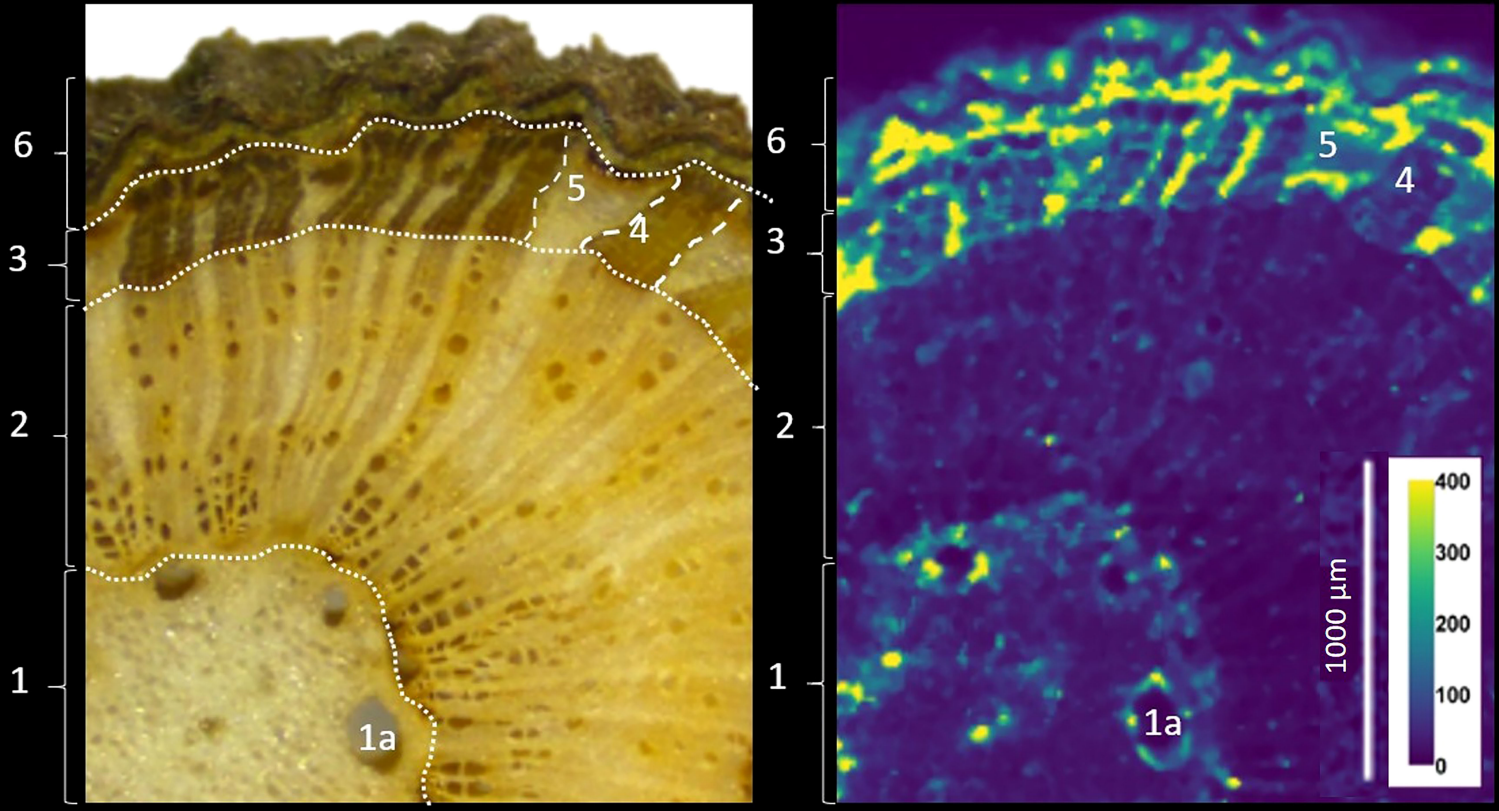
(Left) Microscope image of the branch section, and LA-ICP-MS image for Cd. The intensity is expressed in total counts (cts). From inside to outside: 1: Medulla/pith, including the secretory vessels (1a). 2: wood. 3 to 6: bark, including the phloem part (3) with phloem vessels (4) and phloem rays (5), and the periderm (6), composed of phelloderm and cork. (Right) Map of Cd counts (cts). Cadmium is particularly abundant in the phloem rays (5) and periderm (6).

Cadmium was fourfold more abundant in the bark tissues than in the wood (**Figure 6** and **Table 4**). More specifically, the Cd abundance in the periderm (especially the phelloderm), and the phloem rays was up to seven times higher than in the wood. The phloem rays contained three times more Cd than the phloem vessels. Cadmium was also present in the pith and seemed to be more localized around the lysigenous cavities (i.e. the large channels in the pith).

**Table 4:** Intensity of Cd in the different structures of the branch (T2) and R^2^ of regressions to intensities of other elements detected by LA-ICP-MS.

Tissue	Intensity^$^	R^2 $$^					
	Cd	Ca	Mn	Zn	Cu	Ni	P
**1 Medulla/pith**	2.87	0.32	0.44	0.36	0.12	0.26	0.34
**2 Wood**	1	0.27	0.36	0.40	0.31	0.21	0.32
**3 Bark phloem**	4.40	0.43	0.54	0.48	0.30	0.24	0.45
4 Phloem vessels	2.39	0.37	0.51	0.52	0.28	0.18	0.47
5 Phloem Rays	7.29	0.26	0.41	0.35	0.15	0.15	0.29
**6 Periderm**	7.17	0.13	0.49	0.5	0.24	0.34	0.11

Elements were normalized to ^13^C to for differences in tissue hardness and water content within the sample.

^$^The counts of Cd were normalized to the mean intensity of counts in wood to compare the accumulation of Cd in the different structures of the branch.

^$$^ R^2^ of bivariate fit of Y= log (E/^13^C) and X = log (Cd/^13^C) (with E=Ca, Mn, Zn, Cu, Ni or P). All fits were significant at *p* ≤ 0.05.

Overall, LA-ICP-MS revealed that the nutrients were not evenly distributed in the branch. The living cells of the phloem were richer in nutrients than the dead cells of the xylem (Plomion et al., 2001), and consequently, the wood had generally the lowest intensities whereas the bark and pith were more concentrated in all elements investigated (**Figure 5**; **Figure S7**). The cambial zone was distinguishable as it consists of a few layers of dense, thin-walled cells. The rays in the wood and bark could be visually differentiated too as they have a more concentrated mineral content than the vessels, which is consistent with their role in nutrient translocation. In every compartment Cd, Mn and Zn co-localized strongly, while the distribution of Cd in the tissues correlated less with Ca and P and especially weak correlations for Cu and Ni were found (**Table 4**). In addition, the correlations were weaker in the wood (and thus xylem) tissues, while in the bark, and especially in the phloem vessels the elements correlated highly. Cadmium accumulated more in the phloem rays than in the vessels compared to the nutrients. In the periderm, Cd strongly correlated with Zn and Mn (R^2^ = 0.50), while the correlations with P and Ca were weak (R^2^<0.15).

## Discussion

### High soil to plant transfer of Cd in cacao

The chosen cacao cultivar efficiently took up Cd from the soil as indicated by the leaf-soil transfer factor (TF_leaf IF2-soil_ = 12.5). A survey in Ecuador in cacao plantations (*n*=560) revealed an average TF_leaf-soil_ of 4.5 (Argüello et al., 2019). Likewise, compared to other woody species, TF_leaf-soil_ values were higher in our study. Willow and oak showed a lower transfer of Cd to foliar tissues compared to cacao (Sevel et al., 2009; Van Slycken et al., 2013), although in poplar a similar transfer efficiency for Cd has been described (Laureysens et al., 2004). The high transfer of Cd from soil to plant in our study can be partly explained by the low pH and the low % SOC of the soil that increases the soil Cd bioavailability (Smolders and Mertens, 2013). In addition to these soil properties, the high Cd accumulating properties of the chosen cultivar may have exacerbated the Cd concentrations in the nibs (2 mg Cd kg^-1^). Together, the properties of the soil and the cultivar led to a high Cd concentration in the cacao beans that exceeded the typical trade limits of 0.6 mg Cd kg^-1^.

Cadmium isotope fractionation was used to gain more information on the Cd uptake mechanisms of the cacao tree. The Cd in the cacao plant was lighter than in the Ca(NO_3_)_2_-extractable soil pool (**Figure 1** and **Table 3**), indicating that preferentially light Cd isotopes were taken up from the soil. The isotope fractionation found in this study was similar to previous studies [Δ^114/110^Cd_plant-source_ 0.00 to -0.45‰ (Wiggenhauser et al., 2022)], even though these values origin not only from cacao and were obtained in soils with distinct soil properties, including soil pH. The similar isotope fractionation for plant Cd uptake in previous studies and our acidic soil-cacao system suggests that Cd uptake mechanisms may be very similar in distinct soil-plant systems. Currently, it is suggested that the major processes that induce Cd isotope fractionation during plant uptake are Cd complexation in solution by e.g., dissolved organic matter (Ratié et al., 2021), sorption to the apoplast (Wiggenhauser et al., 2021b), and cross membrane transport (Moore et al., 2020). Given the low pH in our study, other potential processes that fractionate Cd in the soil-plant interface such as complexation in the solution and sorption in the apoplast should be negligible (Degryse et al., 2009). Consequently, TcNRAMP5, a Mn-transporter identified for Cd uptake in cacao (Ullah et al., 2018) that preferentially takes up light isotopes (Moore et al., 2020) may be major driver for the preferential uptake of light Cd isotopes in our study.

### Limited retention of Cd in the transfer from root to the aerial tissues

**Figure 7:**
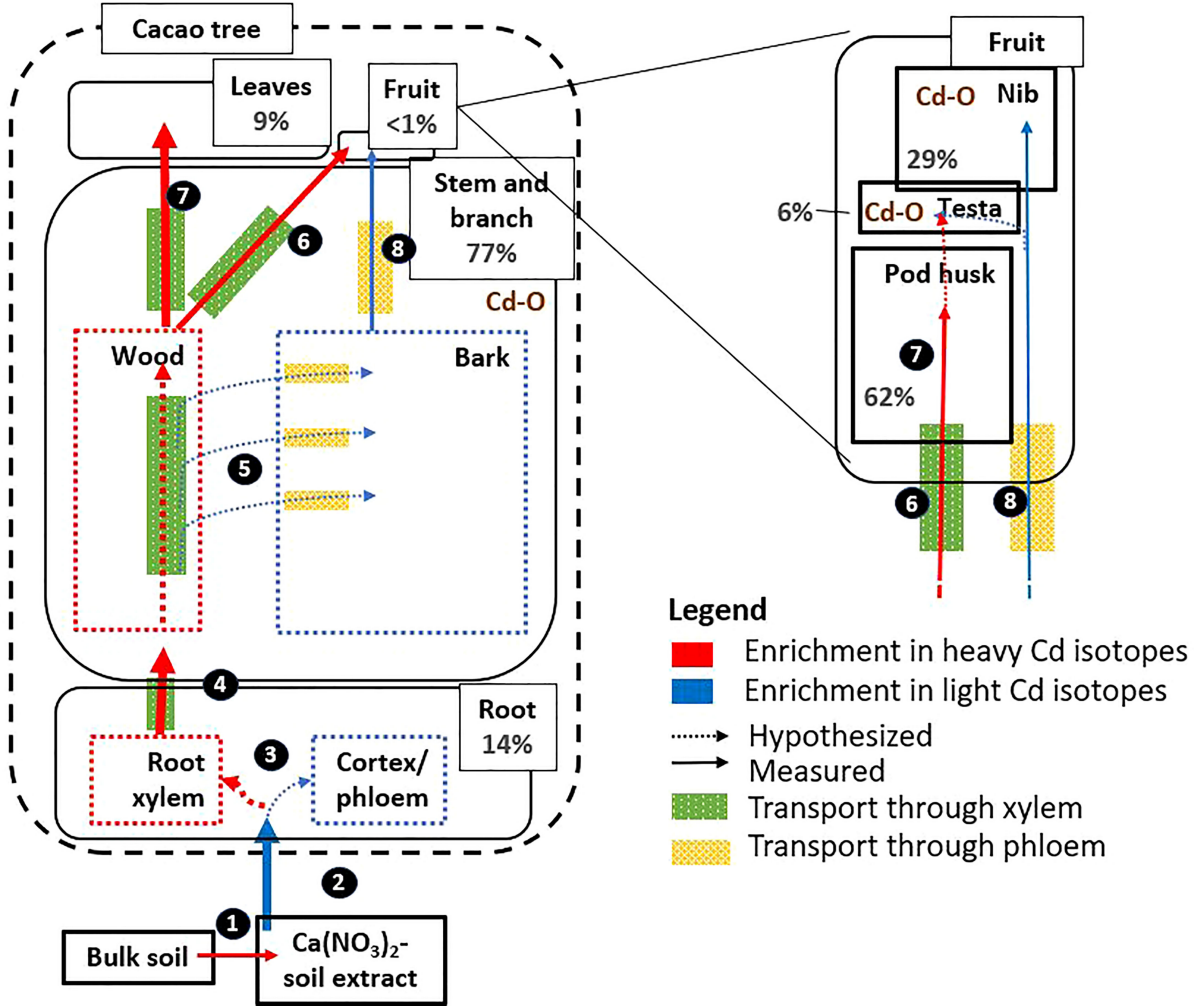
Proposition of Cd pathways, in high Cd accumulator NA 312 of cacao. The figure includes information on Cd mass balance (% in grey, size of the boxes and arrows are indicative to the main Cd fractions and transport rates), Cd isotope fractionation, and Cd speciation. 1) Desorbed Cd was isotopically heavier than adsorbed Cd. 2) The uptake of Cd^2+^ inside the plant favors light isotopes, most likely through cross membrane transport with the protein TcNRAMP5 (Moore et al., 2020). 3) Cd was weakly retained in roots, and the majority was loaded in the xylem for further transport to the shoot, together with nutrients. 4) Preferentially heavy Cd isotopes were transported from root to shoot *via* the xylem. 5) In the branch, Cd was horizontally transferred from the xylem to the phloem in the branches. Lighter Cd isotopes accumulated mostly in the bark (phloem vessels and periderm), while heavier Cd isotopes remained in the xylem. In the branches, Cd was bound to carboxyl ligands, probably to organic acids and/or cell wall components (Cd-O). 6) Cd that accumulated in the branches (i.e. wood and bark), was transported directly to the fruits by both xylem and phloem. 7) Leaves and pod husks received heavier Cd isotopes from the progressive retention of light Cd isotopes in the branches. 8) The nibs were isotopically lighter than the pod husks due to transfer of lighter Cd from the phloem into the nibs. The testa received Cd from both xylem and phloem. The nib was a phloem sink where the majority of Cd was complexed to Cd-O, with phytate as the most plausible ligand. In the testa, Cd was complexed to Cd-O, and more likely to cell wall components or organic acids. Figure layout based on (Kiczka et al., 2010).

The transfer of Cd from root to aerial tissues was high (ITF_leaf-root_≈1, **Table 2**), as observed in other studies on cacao plantations (Vanderschueren et al., 2021). The isotope fractionation revealed that the cacao surface roots in this study were enriched in light isotopes compared to above-ground parts (**Figure 1**), as observed in wheat, barley, rice, cacao, and a Cd accumulator plant (*Solanum nigrum*) (Δ^114/110^Cd _shoot-root_ 0.00 to 0.50 ‰ (Wiggenhauser et al., 2022)). Other crops such as cereals translocate much less Cd from root to shoot (ITF_root-shoot_<0.05) (McLaughlin et al., 2011). In the roots of cereals, Cd is separated from nutrients as a protective measure to reduce the translocation of Cd to other tissues (Wiggenhauser et al., 2021a). Briefly, during the radial transport towards the xylem, Cd is sequestered in vacuoles by the Heavy Metal transporting ATPase 3 (HMA3) influx transporter (Wang et al., 2019) and subsequently bound to S-containing peptides forming stable complexes (Wiggenhauser et al., 2021b). Both processes have been proposed to drive isotope fractionation in the transfer from root to shoot in cereals. Vacuolar sequestration was also suggested to drive Cd fractionation from root to shoot in a contaminated hydroponic system with cacao seedlings (Moore et al., 2020). However, the low retention in the roots of the cacao trees of our study indicates that vacuolar sequestration is rather employed to a small extent to detoxify Cd. Additionally, the observed smaller or similar bulk concentration ratio of Zn, Mn, Ca and Mg relative to Cd between roots and branches, suggests that there is no preferential sequestration of Cd in the roots compared to these nutrients (**Figure 2**). Taken together, we conclude that a small fraction of light Cd was retained in the surface roots and that the majority of Cd was loaded into the xylem for further transport to the shoot together with nutrients (**Figure 7**).

### Branches are the main accumulation compartment for Cd

The branches had the highest Cd concentration (**Table 2**) and stored 57% of the total Cd of the cacao tree (**Figure S3**). Similarly, (Engbersen et al., 2019) reported higher Cd concentration in the branches of cacao trees compared to the other tissues. This contrasts with other Cd-accumulating trees like willow, birch, and oak, where Cd concentrations were 2-3 times higher in foliage than in stems (Evangelou et al., 2012). Cacao exploits thus a different strategy in the partitioning of Cd than these trees, and stores the majority of Cd in its woody parts. The imaging in our study revealed that Cd was mostly accumulating in the bark and more specifically in the phloem rays and the periderm of the branches (**Figure 5**). Indeed, bark concentrations of trace metals are reported to be consistently greater than concentrations in the wood (Pulford and Watson, 2003). Additionally, we observed that in the phloem rays there was a relative enrichment of Cd compared to the Zn and Mn, indicating that Cd was translocated more to the phloem rays, while Zn and Mn were translocated more by the xylem to the foliage (**Figure 2**). Hence, Cd seems to be partially separated from nutrients in the branches.

The fact that Cd isotopes in the leaves were heavier than in the branches may indicate a progressive retention of light isotopes in the branches. In cacao seedlings, light isotopes were retained in the roots and stem compared to the leaves (Moore et al., 2020). The retention of light isotopes in our study probably occurred in the phloem rays and phelloderm of the branches, since these were the main compartments for Cd accumulation (**Figure 6** and **Table 4**), governing the isotopic composition of the bulk measurement. Based on the bulk XANES results, Cd was likely bound to O (C)-ligands in these tissues. This could be due to the complexation of Cd with the O-functional group of an organic ligand and/or cell wall components, as observed in Cd hyperaccumulating species like *Arabidopsis halleri* (Huguet et al., 2012) and *Sedum alfredii* (Tian et al., 2017). Taken together, light Cd was probably separated from heavy Cd and other nutrients like Mn and Zn in its transport from xylem to phloem rays and phelloderm where it finally complexed with an O (C)-ligand (**Figure 7**).

### Differential partitioning of Cd between nibs and leaves

The leaf Cd concentrations were more than two times higher than the bean Cd concentrations (nib< IF2 leaf – IF3 leaf< branch, **Table 2**). This trend followed the general consensus that plants accumulate more Cd in leaves than in seeds (Clemens and Ma, 2016). Indeed, other studies in cacao show leaf Cd concentrations to be higher than the corresponding bean Cd concentrations (Vanderschueren et al., 2021). The mechanism in cacao that affects the partitioning of Cd into reproductive parts seems to be genetically controlled since the variation in bean Cd content has been shown to be much larger than the leaf Cd content (Lewis et al., 2018). In our study, the higher variation in isotope composition observed in fruit tissues compared to vegetative tissues (**Table 2**) may be due to the sampling of the fruits at different maturation. However, the small sample size did not allow to conclude on any effect of maturation on isotope signature.

In the present study, nibs (seeds) were isotopically lighter than leaves (**Table 3**). This result is consistent with a previous study on cacao plants in the field (Barraza et al., 2019). An opposite fractionation was observed in cereals, where Cd in the seeds were isotopically heavier than in the leaves (Imseng et al., 2019; Wiggenhauser et al., 2021a; Zhong et al., 2022). It was suggested that heavy Cd isotopes were favorably transported from xylem to phloem and finally to the seeds (Zhong et al., 2022). In cacao, another mechanism seemed at stake which may be related to the cauliflorous properties of the tree. The similar isotopic composition of the branches and fruits (**Figure 1**) alluded to a more direct transfer of Cd from the branches to the fruits. As suggested before, in the branch, heavier isotopes were probably transferred to the phloem rays. Cadmium that accumulated in the stem and the branches, may thus be transported directly to the fruits by both xylem and phloem, while the leaves receive the heavier Cd in the xylem from the progressive retention of light isotopes in the stem (**Figure 7**).

### Allocation of Cd inside the cacao fruit

Fruits were estimated to be only a small sink of Cd in the tree (<1%, **Figure S3**), and within the fruit the Cd was mostly divided between the nibs and the pod husk (**Figure S4**). It has been suggested that different nutrient distribution pathways exist between cacao pod husks and nibs (De Araujo et al., 2020). The main transport pathway for minerals to the nib has been reported to be the phloem. In contrast, it was suggested that the xylem was more important for the import of nutrients in the testa and the mucilaginous pulp (Humphries, 1944).

The Cd isotope fractionation between the nib and pod husk (**Table 3**) and different nutrient distribution (**Figure 2**) was consistent with this finding. The isotopic composition of the pod husk was in between that of the leaves and the branches (**Figure 1**). This result indicates that the pod husk received heavier Cd from the xylem, although the fractionation is less pronounced than in the leaves. The high K content in the pod husk corroborates the indications that Cd was transferred *via* xylem to the pod husk, since the K comprises 70% of all nutrients in the cacao xylem (De Almeida and Valle, 2007). For the testa, Cd had a similar isotope composition and speciation as the branches and could thus be fed by the xylem and phloem from the branch. Hence, we suggest that Cd was mainly transported from the branches to the cacao fruit, and not remobilized from the leaves to the nibs. The nibs were lighter in isotopes due to the transfer of light isotopes from the phloem, while the pod husk received heavier isotopes from the xylem and the testa received Cd by both xylem and phloem (**Figure 7**).

### Cadmium speciation in the nibs and its implications on bioaccessability

The nibs had a five times higher P/Cd ratio than other tissues (**Table 1** and **Figure 2**). About 85% of the phosphorus measured in the nibs was in the form of phytate. The phytate/Cd and phytate/(Cd+Zn+Fe) molar ratio showed that phytate could be an abundant ligand for Cd. Together with the XANES result of binding of Cd with mostly O-ligands, and probably more specifically O (P)-ligands (**Figure 4**), we suggest that phytate is the most plausible ligand for Cd in the nibs. Information on the role of P and phytate in cacao nibs is scarce. Mounicou et al., 2003 found that in cacao powders up to 20% of Cd was removed after treatment with phytase, indicating that phytate may be an important chelator of Cd. In wheat kernels (seeds), it has been shown that also about 85% of the phosphorus (P) is stored as phytate (Lemmens et al., 2018) and that micronutrients like Fe and Zn can strongly bind to phytate, inhibiting the human absorption of these minerals. The same principle may apply to Cd (Schlemmer et al., 2009), and may consequently have important implications on Cd absorption in human nutrition. However, speciation data in cereal grains currently indicates mostly a binding of Cd to S-ligands, despite the high phytate content (Gu et al., 2020; Wiggenhauser et al., 2021a). It should be noted that these speciation measurements were in systems with higher seed Cd concentrations, which could have affected Cd speciation (Andresen et al., 2013).

### Implications and outlook

We combined for the first time total elemental concentrations, Cd stable isotope analyses, speciation, and imaging in cacao trees grown in field conditions at environmentally relevant soil Cd concentrations. We highlighted that compared to annual crops, the retention of Cd in the roots was limited. Cadmium accumulated mainly in the branches and more specifically in the phloem rays of the bark phloem where it was bound to carboxylic ligands. The weight of evidence suggests that in the high Cd accumulating cacao cultivar NA 312, Cd was transferred like other nutrients from root to shoot and accumulated in the phloem rays and phelloderm of the branches to reduce the transfer to foliage. The main contribution of Cd in the nibs came from the phloem tissues of the branch rather than from the remobilization of the leaves. Finally, in the nibs, Cd was stored by chelation with phytate.

Some limitations affected this study. *Theobroma cacao* L. is a perennial tree with a deep rooting system and evidently sampling in field conditions was constrained. For instance, the surface root samples collected may not represent the whole rooting system. Similarly, the thin branch samples may not represent the bigger branches and stem structures, and the mass balance had to be estimated based on literature. Further, this study was only limited to a high Cd accumulating cultivar, which was deficient in K and P. More comparative studies on other genotypes and hybrids used agronomically are indispensable to extrapolate findings on different control points. We also suggest more speciation measurements as the low Cd concentrations touched the detection limits of XAS, which reduced the sample throughput severely. Despite its limitations, our study has important implications. We extended the limited knowledge on Cd accumulation in perennial, woody crops and revealed that the Cd pathway is markedly different than in annual crops, like the thoroughly studied cereals. The branch was identified as a major tissue for the regulation of the loading of Cd to the nibs. Additional studies which identify the corresponding regulating genes will allow breeding programs to produce low Cd accumulating cultivars in the long term. Grafting has been proposed as a short-term strategy where economically interesting scions are grafted on low cadmium accumulating rootstocks. However, as the scion may be the main regulator of Cd loading into the nibs, further studies must be conducted to validate the efficacy of this strategy. Postharvest mitigation research may benefit from the knowledge of the chemical speciation of Cd in nibs and testa as this is a first step in grasping the chemical processes behind the mobilization of Cd within cacao nibs observed during fermentation. A final piece of important information is the chelation of Cd with phytate which may reduce the absorption of Cd in humans and calls for follow-up studies on human absorption of Cd derived from cacao products.

## Data availability statement

The raw data supporting the conclusions of this article will be made available by the authors, without undue reservation.

## Author contributions

HB contributed to the conceptualization, methodology, investigation, validation, formal analyses, investigation, writing- original draft. A-MA, MW, PU, and ES contributed to the conceptualization, methodology, investigation, writing–review, and editing. PT, SC, CM, JB, GL, DT, SP, and CL contributed to the methodology, investigation, and writing-review. GS contributed to the conceptualization, methodology, validation, formal analyses, investigation, data curation, visualization, writing–review and editing, and funding acquisition. All authors contributed to the article and approved the submitted version.

## Funding

This work was financially supported by the French National Research Agency program ‘Investissements d’avenir’ (ANR-15-IDEX- 02), and in the CNRS/INSU/EC2CO project CACAO. HB and GS (ISTerre) are part of Labex OSUG (ANR10 LABX56). GS, HB and ES are working in the framework of the Program Hubert Curien “TOURNESOL” 2020–2021 (project n° 44274TC). HB, MW, ES, and MW are members of the COST Action CA19116 PLANTMETALS (COST, European Cooperation in Science and Technology, www.cost.eu). Funding for the LA-ICP-MS was obtained from the Special Research Fund (BOF) of KU Leuven with project AKUL/19/007 – ZKD8131.

## Acknowledgments

We express our gratitude to the entire staff of the Cocoa Research Centre for the help with the collection of samples. We thank the staff of the Geochemistry Mineralogy Platform at ISTerre, of KULeuven and ENS Lyon for their laboratory support. We acknowledge the review committees for the provision of beamtime at SAMBA (Soleil) and BM30 (ESRF) and we thank the beamline staff on both beamlines for their help during measurement. We thank Murray McBride for providing the Cd/Ca oxalate compounds. Lastly, we also thank the members of the COST Action CA19116 PLANTMETALS.

## Conflict of interest

The authors declare that the research was conducted in the absence of any commercial or financial relationships that could be construed as a potential conflict of interest.

## Publisher’s note

All claims expressed in this article are solely those of the authors and do not necessarily represent those of their affiliated organizations, or those of the publisher, the editors and the reviewers. Any product that may be evaluated in this article, or claim that may be made by its manufacturer, is not guaranteed or endorsed by the publisher.
