# Supplementary material for: From soil to cacao bean: Unravelling the pathways of cadmium translocation in a high Cd accumulating cultivar of Theobroma cacao L
Source: Front Plant Sci. 2022 Dec 2;13:1055912. doi: 10.3389/fpls.2022.1055912 (PMC9755593; doi:10.3389/fpls.2022.1055912)
Supplement: Supplementary file 1 [file DataSheet_1.docx]

Supplementary Material

From soil to cacao bean: Unravelling the pathways of cadmium translocation in a high Cd accumulating cultivar of *Theobroma cacao* L.

Hester Blommaert^1*^, Anne-Marie Aucour^2^, Matthias Wiggenhauser^3*^, Claudia Moens^4^, Philippe Telouk^2^, Sylvain Campillo^1^, Jacques Beauchêne^5^, Gautier Landrot^6^, Denis Testemale^7^, Serge Pin^8^, Caleb Lewis^9^, Pathmanathan Umaharan^9^, Erik Smolders^4^, Géraldine Sarret^1^

^1^Université Grenoble Alpes, Université Savoie Mont Blanc, CNRS, IRD, Université G. Eiffel, ISTERRE, Grenoble, France

^2^Université de Lyon, Université Lyon 1, ENS de Lyon, CNRS, UMR 5276 LGL-TPE, F-6922, Villeurbanne, France

^3^Institute of Agricultural Sciences, ETH Zurich, Eschikon 33, CH-8315, Lindau, Switzerland

^4^Division of Soil and Water Management, Department of Earth and Environmental Sciences, KU Leuven, Belgium

^5^CIRAD, UMR Ecologie des Forêts de Guyane (EcoFoG), AgroParisTech, CNRS, INRA, Université des Antilles, Université de Guyane, 97310, Kourou, France

^6^Synchrotron SOLEIL, L’Orme des Merisiers, Saint-Aubin, BP 48 91192, Gif-sur-Yvette, France

^7^Univ. Grenoble Alpes, CNRS, Grenoble INP, Institut Néel, 38000, Grenoble, France

^8^Université Paris-Saclay, CEA, CNRS, NIMBE, 91191, Gif-sur-Yvette, France

^9^Cocoa Research Centre, University of the West Indies, St. Augustine, Trinidad and Tobago

*** Correspondence:**Hester Blommaert
[hester.blommaert@univ-grenoble-alpes.fr](mailto:hester.blommaert@univ-grenoble-alpes.fr)

Matthias Wiggenhauser
matthias.wiggenhauser@usys.ethz.ch

# Principle and procedure phytate analyses

The phytate content in the nibs was determined in triplicate with a phytic acid assay (McKie and McCleary, 2016). Twenty mL 0.66 M HCl was added to ground cacao nibs (500 mg ± 20 mg) and incubated for 16h in an end-over-end shaker (150 rpm, 20°C). The extract was centrifuged, and the supernatant was neutralized by addition of NaOH (0.75 M). Two aliquots were taken from the supernatant, one for a ‘total P’ reaction and one for the ‘free P’ reaction. In the total P, an excess of phytase and alkaline phosphatase was added, for the breakdown of polyphosphates to monophosphate. Phosphate was measured in both aliquots colorimetrically by adding a color reagent solution consisting of ammonium molybdate, ascorbic acid, and sulfuric acid (Murphy and Riley, 1962) [measurement at a wavelength of 655 nm using a UV/VIS spectrophotometer (Lambda 25, PerkinElmer, Waltham, MA, USA)]. The concentration of bound P in the supernatants was obtained by subtracting free P concentration from the total P concentration. The bound P was then converted to the amount of phytate under the assumption that the measured P released from enzymatic hydrolysis exclusively originates from phytic acid.

# 2 Supplementary Figures and Tables

**Table S1:** Quality control of elemental analyses. The measured concentration, certified and indicative values are expressed in mg kg^-1^ DW^-1^. Fe and Cr showed poor recovery results and were not considered in further analyses.

| **Sample type** | **Description** |  |  | **K** | **Ca** | **Cr** | **Cu** | **Mn** | **Ni** | **Pb** | **Zn** | **Cd** | **P** | **S** | **Mg** | **Fe** |
| --- | --- | --- | --- | --- | --- | --- | --- | --- | --- | --- | --- | --- | --- | --- | --- | --- |
| Soil TM3 | Internal KULeuven soil standard (n=4)^c^ | Indicative value | | 2518.70 | 2125.60 | 26.17 | 11.10 | 441.64 | 12.31 | 19.36 | 44.81 | 0.31 | 559.82 | - | 2291.65 | 16465.45 |
|  |  | Measured conc. | | 2505.24 | 2096.63 | 26.06 | 10.67 | 432.80 | 12.58 | 18.38 | 42.16 | 0.28 | 501.94 | 119.29 | 2377.50 | 16007.59 |
|  |  | SD^a^ |  | 38.25 | 50.77 | 1.01 | 0.53 | 30.74 | 0.71 | 0.78 | 4.29 | 0.03 | 20.83 | 0.39 | 40.34 | 439.92 |
|  |  | **Recovery digestion (%)^b^** | | **99** | **99** | **100** | **96** | **98** | **102** | **95** | **94** | **91** | **90** | **-** | **104** | **97** |
| Baking chocolate | NIST 2384 (n=2)^c^ | Certified value | | 8650.00 | 840.00 | - | 23.90 | 20.80 | - | - | 37.60 | 0.07 | 3330.00 | **-** | 2610.00 | 132.00 |
|  |  | Measured conc. | | 8666.07 | 899.61 | 0.71 | 24.08 | 20.30 | 6.07 | - | 36.13 | 0.07 | 3368.30 | 1428.44 | 2702.26 | 22.32 |
|  |  | SD^a^ |  | 46.99 | 22.29 | 0.04 | 0.20 | 0.16 | 0.59 | - | 0.22 | 0.00 | 26.48 | 10.69 | 20.39 | 0.63 |
|  |  | **Recovery digestion (%)^b^** | | **100** | **107** | **-** | **101** | **98** | **-** | **-** | **96** | **89** | **101** | **-** | **104** | **17** |
| Tomato leaves | NIST 1573a (n=2)^c^ | Certified value | | 27000.00 | 50500.00 | 1.99 | 4.70 | 246.00 | 1.59 | - | 30.90 | 1.52 | 2160.00 | 9600.00 | 12000.00 | 368.00 |
|  |  | Measured conc. | | 26855.00 | 50664.44 | 1.65 | 4.76 | 238.60 | 1.61 | 0.52 | 28.72 | 1.42 | 2321.52 | 9903.57 | 11121.44 | 310.99 |
|  |  | SD^a^ |  | 469.30 | 700.35 | 0.00 | 0.04 | 2.35 | 0.18 | 0.00 | 0.07 | 0.02 | 27.55 | 163.37 | 199.93 | 3.71 |
|  |  | **Recovery digestion (%)^b^** | | **99** | **100** | **83** | **101** | **97** | **102** | **-** | **93** | **94** | **107** | **103** | **93** | **85** |

^a^SD= standard deviation of the mean
^b^equal to [measured concentration]/[reference concentration]*100
^c^number of processing replicates (individually digested, and measured)

**Table S2:** Quality control of Cd isotope measurements

| **Sample Type** | **Description** | **Reference Cd conc** | | **Measured Cd conc^a^** | | | **Recovery digestion^b^** | **Purification yield^c^** | | **Ref Cd isotope ratio** | | **Measured Cd isotope ratio** | | |
| --- | --- | --- | --- | --- | --- | --- | --- | --- | --- | --- | --- | --- | --- | --- |
|  |  | mg (kg DW)^-1^ | | mg (kg DW)^-1^ | | | % | % | | δ^114/110^Cd (‰) | | δ^114/110^Cd (‰) | | |
|  |  | mean | sd | mean | sd | n^d^ | mean | mean | sd | mean | sd | mean | sd | n |
| white cabbage | BCR-679 | 1.66 | 0.07 | 1.70 | 0.06 | 6 | 101 | 100 | 20 | 0.22^e^ | 0.02 | 0.18 | 0.03 | 10 |
|  |  |  |  |  |  |  |  |  |  |  |  |  |  |  |
| soil | NIST S Joaquim | 0.36 | 0.01 | 0.35 | 0.07 | 4 | 93 | 89 | 5 | -0.22 | 0.02 | -0.22 | 0.04 | 4 |
|  |  |  |  |  |  |  |  |  |  |  |  |  |  |  |
| Münster Cd | isotope standard | - | - | - | - | - | - | - |  | 4.49^f^ | 0.05 | 4.43 | 0.06 | 5 |
|  |  |  |  |  |  |  |  |  |  |  |  |  |  |  |

^a^measured in unpurified samples,
^b^equal to [measured concentration]/ [reference concentration]*100,
^c^equal to [concentration purified sample]/[concentration non purified sample]*100,
^d^number of processing replicates (individually digested, purified for isotope analyses, and measured for concentrations and isotope ratios),
^e^value obtained by Wiggenhauser et al. 2021,
^f^values obtained by Cloquet et al. 2005, Pallavicini et al. 2014 and Liu et al. 2019.

**Table S3:** Calculation of Cd mass balances for cacao trees

| **Tissue** | **Replicate** | **N**** | **Mass** | **Mass fraction** | **Total Cd** | **Cd fraction** |
| --- | --- | --- | --- | --- | --- | --- |
|  |  |  | **kg** |  | **mg** |  |
| Roots | T2 | 1 | 31.50^$^ | 0.24 | 86.17 | 0.14 |
|  | T4 | 1 | 31.50^$^ | 0.24 | 84.15 | 0.13 |
|  | T6 | 1 | 31.50^$^ | 0.24 | 132.84 | 0.18 |
| Stem^#^ | T2 | 1 | 22.50^$^ | 0.17 | 130.98 | 0.21 |
|  | T4 | 1 | 22.50^$^ | 0.17 | 132.98 | 0.21 |
|  | T6 | 1 | 22.50^$^ | 0.17 | 148.92 | 0.20 |
| Branch | T2 | 1 | 61.90^$^ | 0.47 | 360.34 | 0.58 |
|  | T4 | 1 | 61.90^$^ | 0.47 | 365.83 | 0.57 |
|  | T6 | 1 | 61.90^$^ | 0.47 | 409.70 | 0.56 |
| Leaves IF2 | T2 | 1 | 7.75^$^ | 0.06 | 23.40 | 0.04 |
|  | T4 | 1 | 7.75^$^ | 0.06 | 36.96 | 0.06 |
|  | T6 | 1 | 7.75^$^ | 0.06 | 20.77 | 0.03 |
| Leaves IF3 | T2 | 1 | 7.75^$^ | 0.06 | 22.47 | 0.04 |
|  | T4 | 1 | 7.75^$^ | 0.06 | 27.07 | 0.04 |
|  | T6 | 1 | 7.75^$^ | 0.06 | 22.33 | 0.03 |
| Pod husk | T2 | 1 | 0.027* | <0.01 | 0.10 | <0.01 |
|  | T4 | 2 | 0.023* | <0.01 | 0.07 | <0.01 |
|  | T6 | 2 | 0.024* | <0.01 | 0.06 | <0.01 |
| Placenta | T2 | 1 | 0.001* | <0.01 | <0.01 | <0.01 |
|  | T4 | 2 | 0.001* | <0.01 | <0.01 | <0.01 |
|  | T6 | 1 | 0.001* | <0.01 | <0.01 | <0.01 |
| Testa | T2 | 1 | 0.003* | <0.01 | 0.01 | <0.01 |
|  | T4 | 2 | 0.002* | <0.01 | <0.01 | <0.01 |
|  | T6 | 1 | 0.002* | <0.01 | <0.01 | <0.01 |
| Nib | T2 | 1 | 0.011* | <0.01 | 0.02 | <0.01 |
|  | T4 | 2 | 0.014* | <0.01 | 0.03 | <0.01 |
|  | T6 | 2 | 0.014* | <0.01 | 0.03 | <0.01 |
| **Fruit** | T2 | 1 | 0.042 | <0.01 | 0.13 | <0.01 |
|  | T4 | 2 | 0.04 | <0.01 | 0.10 | <0.01 |
|  | T6 | 2 | 0.045 | <0.01 | 0.10 | <0.01 |
| **Tree** | T2 | 1 | 131.44 | 1 | 623.36 | 1 |
|  | T4 | 1 | 131.48 | 1 | 646.99 | 1 |
|  | T6 | 1 | 131.47 | 1 | 734.56 | 1 |

^$^The masses of the roots, stem, branches and leaves were estimated based on the biomass distribution reported by (Nurafiza *et al.*, 2017).
*The trees in this study were sampled out of cacao peak fruiting season, hence the authors did not consider fruits in the biomass distribution. Therefore, to include the reproductive tissues, the dry weights of the sampled fruits in this study were added to calculate the Cd mass distribution in the total trees.
^#^As no measurement of the stem was done, the same Cd concentration as in the branches was assumed. ** Some trees contained two fruits, others only one.
*Mass fraction = mass _tissue_/mass _tree_Total Cd (mg) = mass _tissue_ (kg) * Cd _tissue_ (mg kg^-1^)
Cd fraction = total Cd _tissue_/total Cd _tree_*

**Table S4**: p-values of statistical tests performed to verify the assumptions of ANOVA and p-value for ANOVA tests.

| X | Y | Shapiro-Wilk | O'Brien | Brown-Forsythe | ANOVA |
| --- | --- | --- | --- | --- | --- |
| Tissue | Cd (mg kg^-1^) | 0.11 | 0.07 | 0.07 | <0.0001 |
| Tissue | δ^114/110^Cd (‰) | 0.14 | 0.29 | 0.46 | <0.0001 |
| Tissue | log (Zn/Cd) (mM) | 0.76 | 0.46 | 0.79 | <0.0001 |
| Tissue | log (Mn/Cd) (mM) | 0.30 | 0.17 | 0.70 | <0.0000 |
| Tissue | log (Ca/Cd) (mM) | 0.21 | 0.62 | 0.91 | <0.0001 |
| Tissue | log (Mg/Cd) (mM) | 0.60 | 0.16 | 0.59 | 0.0014 |
| Tissue | log (S/Cd) (mM) | 0.18 | 0.57 | 0.80 | <0.0001 |
| Tissue | log (K/Cd) (mM) | 0.51 | 0.66 | 0.89 | <0.0001 |
| Tissue | log (Cu/Cd) (mM) | 0.61 | 0.20 | 0.36 | <0.0001 |
| Tissue | log (P/Cd) (mM) | 0.00 | 0.08 | 0.39 | <0.0001 |

**Table S5:** Soil properties of the soil of the accession plot of NA 312 (*n*=3).

|  |  | **Topsoil** |  | **Subsoil** |  |
| --- | --- | --- | --- | --- | --- |
|  |  | mean | SD | mean | SD |
| pH |  | **4.13** | 0.11 | **3.74** | 0.06 |
| Organic Carbon | % | **1.86** | 0.36 |  |  |
| Aqua regia soil Cd | mg/kg | **0.22** | 0.05 | **0.06** | 0.01 |
| Available Cd* | mg/kg | **0.08** | 0.02 | **0.03** | 0.01 |
| Total Cd** | mg/kg | **0.28** | 0.05 |  |  |
| eCEC | cmol_c_/kg | **11.65** | 0.12 | **11.16** | 0.31 |
| Oxalate extractable Al | g/kg | **1.00** | 0.05 | **1.01** | 0.11 |
| Oxalate extractable Fe | g/kg | **4.86** | 0.20 | **3.20** | 0.89 |
| Oxalate extractable Mn | g/kg | **0.83** | 0.11 | **0.45** | 0.22 |

*Ca(NO3)2-extract: 0.05 M Ca(NO_3_)_2_ (5 g soil: 50 mL Ca(NO_3_)_2_)
** Total digestion (aqua regia with subsequent HF digestion)
The soil at the ICGT was previously characterized as a clayey loam soil (Lewis et al., 2018). The measured pH was around the minimum reported in a survey of cacao plantations in Ecuador (4.1-7.9, mean: 6.0) (Argüello et al., 2019). Organic carbon content was on the lower end of the range, while ECEC, oxalate extractable Al, Fe and Mn followed the average trends found in the studied Ecuadorian farms.

**Table S6:** Mean and SD of concentrations of nutrients in the various tissues (DW).

|  | **Zn (mg kg^-1^)** | | | **Mn (mg kg^-1^)** | | | **Ca (g kg^-1^)** | | | **Mg (g kg^-1^)** | | |
| --- | --- | --- | --- | --- | --- | --- | --- | --- | --- | --- | --- | --- |
|  | mean | SD | | mean | SD | | mean | | SD | mean | SD | |
| roots | 46.80 | 10.24 | | 101.10 | 54.52 | | 7.61 | | 1.08 | 3.56 | 2.16 | |
| branch | 139.20 | 25.15 | | 465.10 | 114.25 | | 20.86 | | 8.16 | 5.54 | 2.14 | |
| pod husk | 71.40 | 6.92 | | 308.40 | 134.74 | | 4.98 | | 1.67 | 3.15 | 0.65 | |
| placenta | 33.40 | 4.79 | | 58.70 | 35.09 | | 1.51 | | 0.30 | 1.59 | 0.44 | |
| testa | 36.10 | 6.87 | | 99.70 | 28.09 | | 4.53 | | 1.69 | 2.86 | 0.53 | |
| nib | 46.70 | 4.90 | | 93.60 | 24.77 | | 1.24 | | 0.22 | 3.51 | 0.27 | |
| leaves IF2 | 183.80 | 21.52 | | 1776.90 | 287.47 | | 16.12 | | 2.31 | 8.54 | 0.46 | |
| leaves IF3 | 177.60 | 15.65 | | 1863.60 | 306.21 | | 17.33 | | 1.78 | 8.53 | 0.56 | |
|  | **P (g kg^-1^)** | | | **Cu (mg kg^-1^)** | | | **S (g kg^-1^)** | | | **K (g kg^-1^)** | | |
|  | mean | | SD | mean | | SD | mean | SD | | mean | | SD |
| roots | 0.99 | | 0.78 | 9.80 | | 3.70 | 1.69 | 0.33 | | 5.44 | | 0.87 |
| branch | 1.04 | | 0.40 | 6.50 | | 1.44 | 1.00 | 0.21 | | 10.12 | | 3.82 |
| pod husk | 1.18 | | 0.13 | 10.10 | | 1.59 | 1.27 | 0.08 | | 28.57 | | 5.20 |
| placenta | 1.47 | | 0.10 | 9.60 | | 3.96 | 1.38 | 0.27 | | 15.71 | | 3.12 |
| testa | 0.99 | | 0.17 | 11.50 | | 1.54 | 1.17 | 0.07 | | 10.46 | | 0.37 |
| nib | 4.24 | | 0.53 | 16.80 | | 1.82 | 1.88 | 0.09 | | 9.50 | | 2.19 |
| leaves IF2 | 1.39 | | 0.08 | 4.60 | | 0.25 | 2.32 | 0.09 | | 11.58 | | 2.01 |
| leaves IF3 | 1.17 | | 0.14 | 3.80 | | 0.26 | 1.93 | 0.08 | | 8.35 | | 2.09 |

**Table S7:** R^2^ and F-test for the linear correlation between the log transform of bulk Cd concentrations and nutrients in the various organs (Figure S3), all values measured are shown for the n=3 trees.

| **Element** | **R^2^** | **F(1,28)** | **p-value** | **equation** |
| --- | --- | --- | --- | --- |
| **log Zn** | 0.38 | 17.41 | 0.0003 | 3.49+0.78x |
| **log Mn** | 0.38 | 17.31 | 0.0003 | 3.97+1.54x |
| **log Ca** | 0.62 | 46.52 | <0.0001 | 7.01+1.62x |
| **log Mg** | 0.41 | 19.8 | <0.0001 | 7.55+0.82x |
|  |  |  |  |  |
| **log P** | 0.00 | 1.12 | 0.29 | 7.46-0.27x |
| **log Cu** | 0.10 | 3.22 | 0.08 | 2.45-0.31x |
| **log S** | 0.01 | 0.14 | 0.72 | 7.35-0.038x |
| **log K** | 0.01 | 2.72 | 0.11 | 9.64-0.30x |


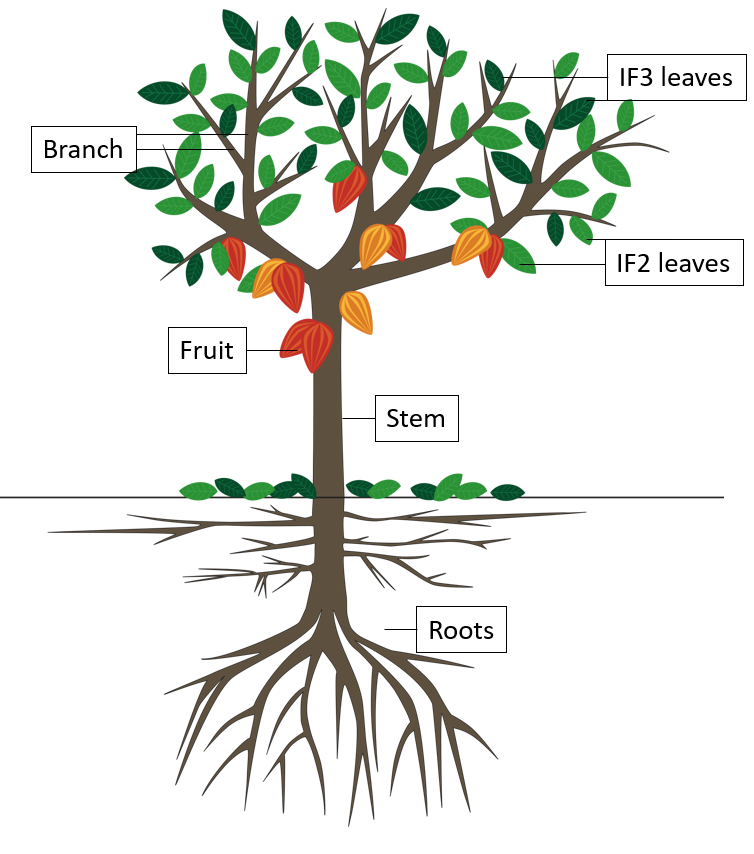

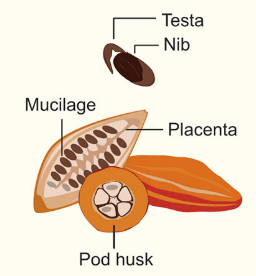


D

C

B

A


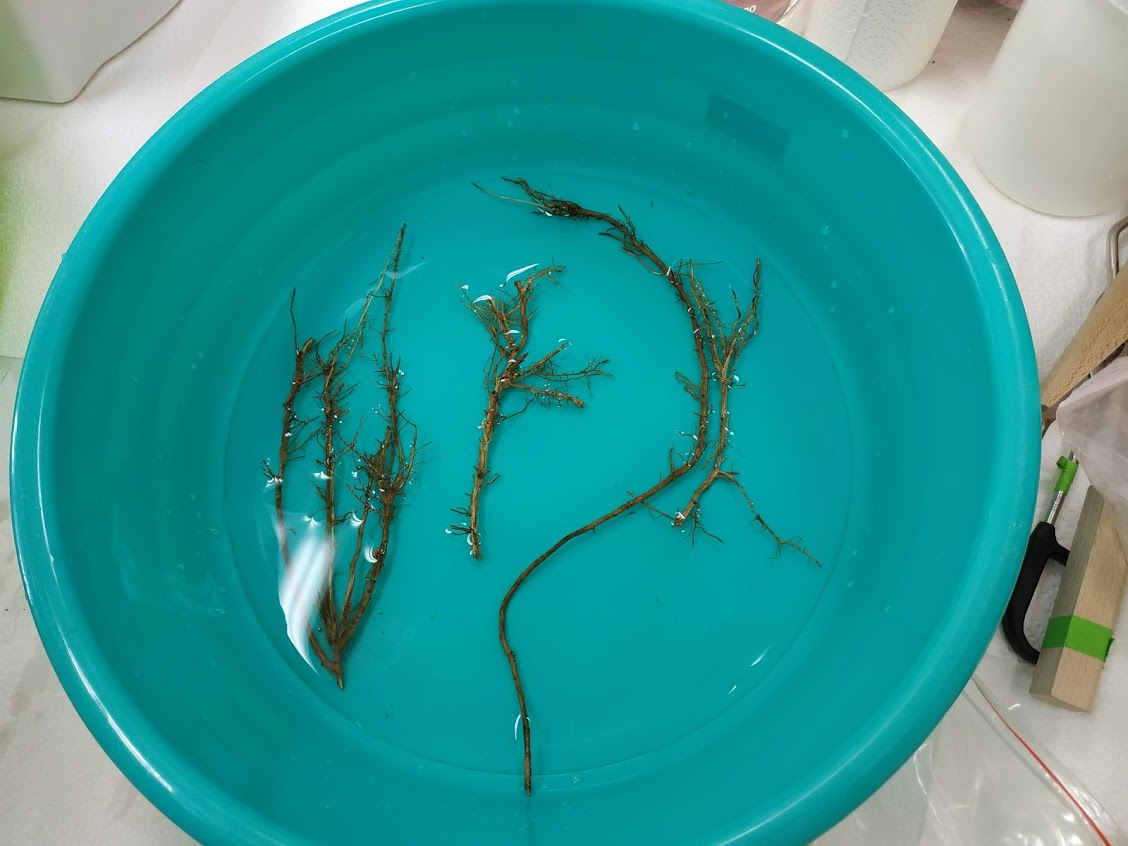

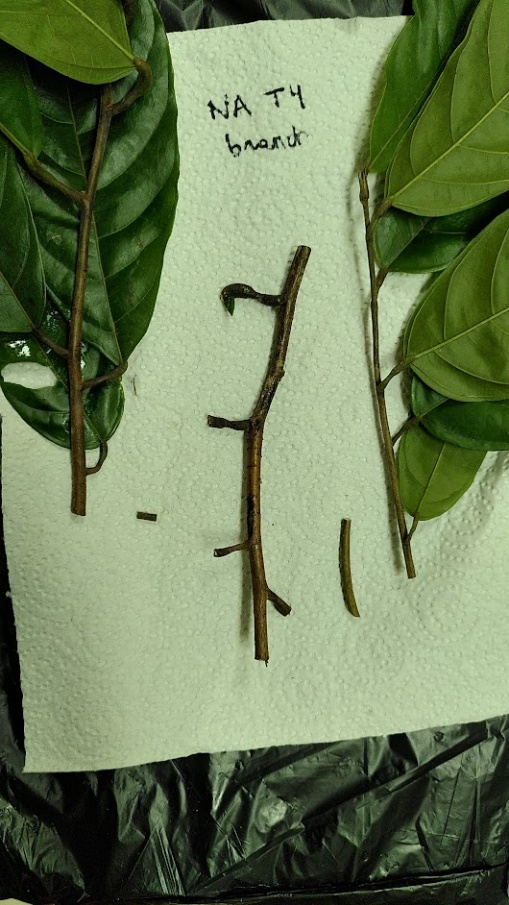


F

E


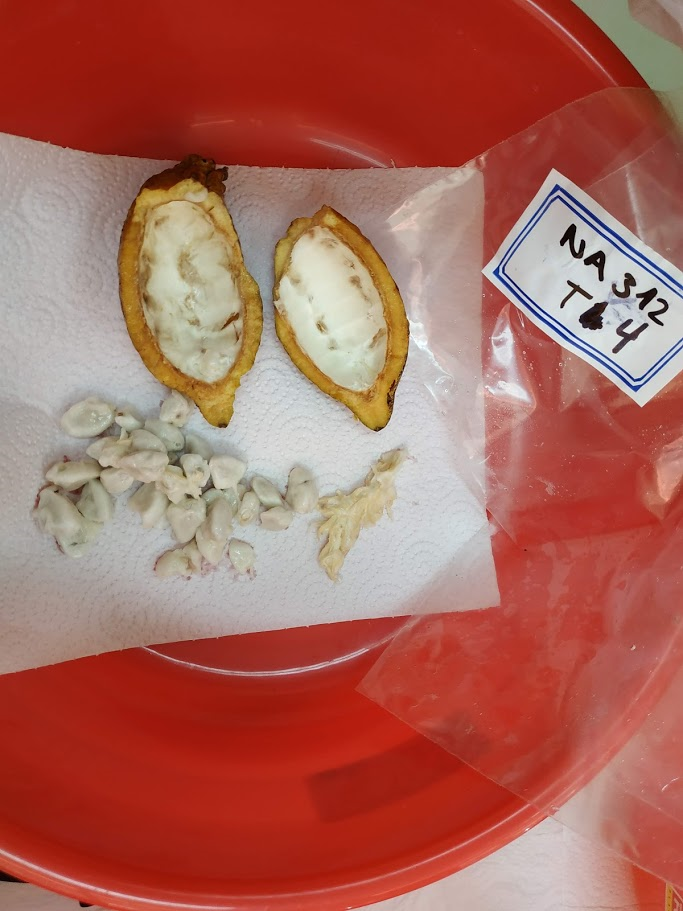

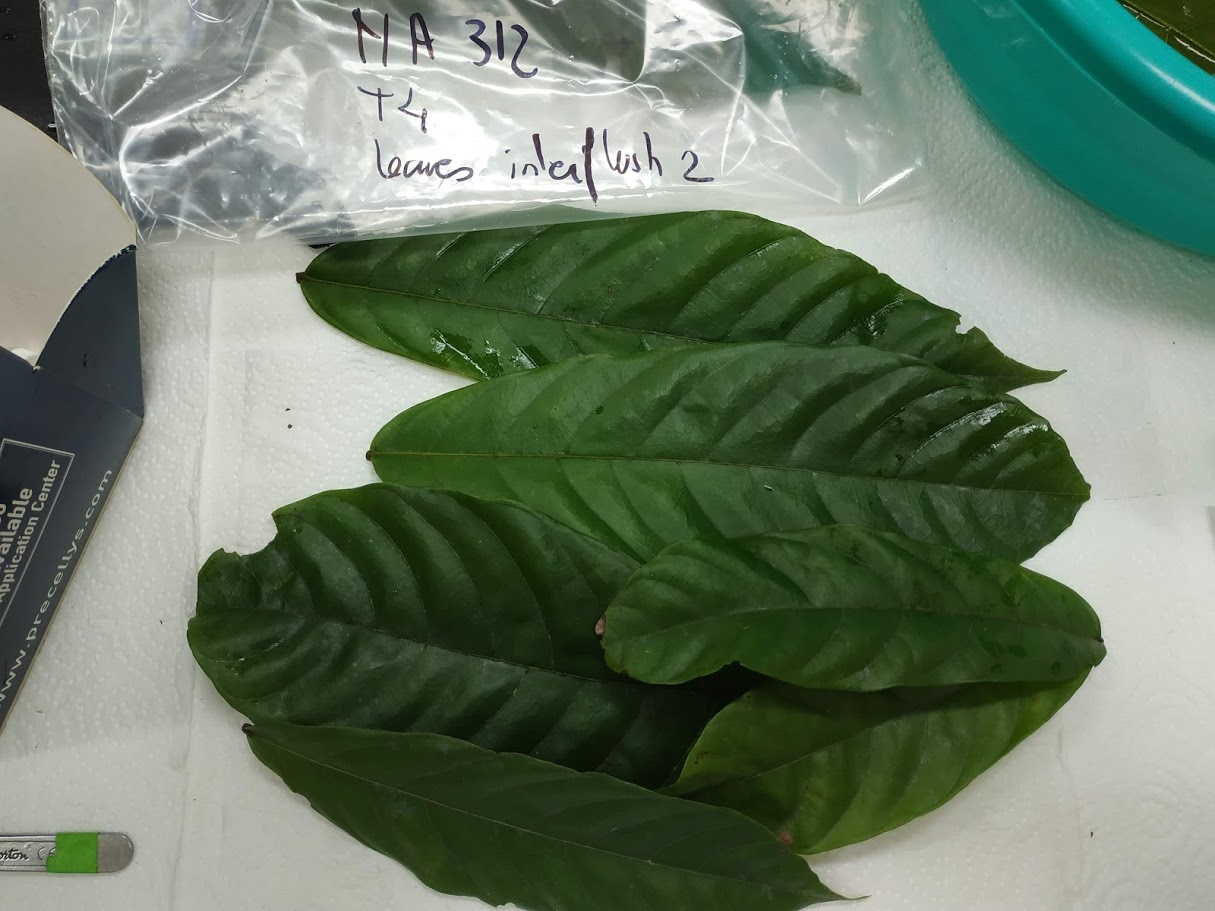


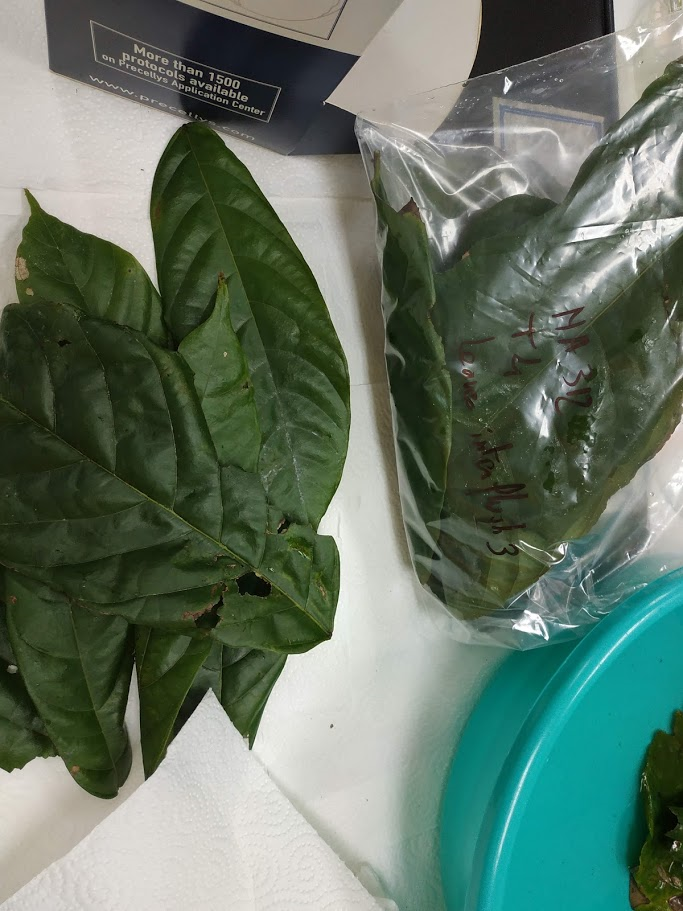


G

**Figure S1:** (A) Terminology of the sampled organs of the cacao tree. (B) Tissues in a cacao fruit (Vanderschueren *et al.*, 2021). The cacao fruit or pod is made up of a large woody outer pod husk, filled with 20–50 cacao beans (seeds). The cacao bean consists of a nib and an outer shell, the testa. The nib is the only part of the cacao bean that is retained during chocolate processing. (C) Example of roots collected (diameter 1-10 mm). (D) Example of branch sampled (diameter 3-7 mm). (E) Example of fruit tissues sampled. (F) Example of IF2-leaves. (G) Example of IF3 leaves.


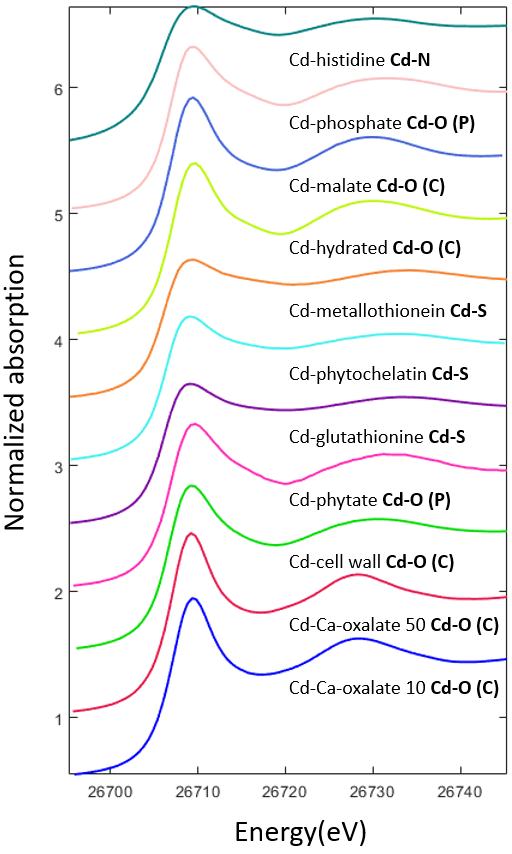


**Figure S2.** Cd K-edge XANES spectra of selected reference materials. A previously recorded database of Cd reference spectra recorded at 15 to 20K was used, containing Cd-cell wall, Cd-glutathionine, Cd-phytochelatin, Cd-hydrated, Cd-malate, Cd-histidine, Cd phosphate. The preparation and measurements of this dataset is described in (Huguet *et al.*, 2012, 2015). As additional standards, Cd-metallothionein extracted from mussels (Bataille *et al.*, 2005), Cd phytate (phytate/Cd molar ratio = 5), Cd-Ca-oxalate (Ca-oxalate/Cd molar ratio = 10 and 50) (McBride *et al.*, 2017) were measured at BM30 and included in the database.


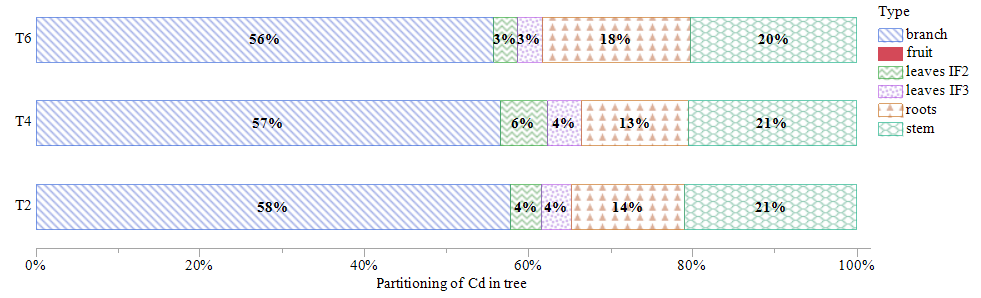


**Figure S3.** Partitioning of Cd in the three trees assuming a tree biomass as described by (Nurafiza *et al.*, 2017). Assumed biomass of the different tissues: roots 31.5 kg, stem: 22.5 kg, branch: 61.9 kg, IF2 leaves: 7.75 kg, IF3 leaves: 7.75 kg, fruit: 0.05 kg


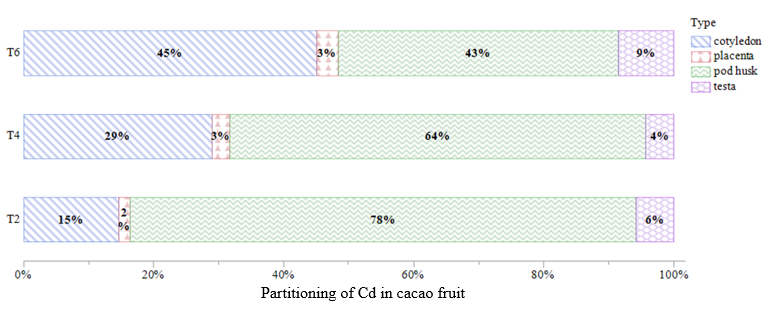


**Figure S4:** Partitioning of Cd in the cacao fruit of NA312.


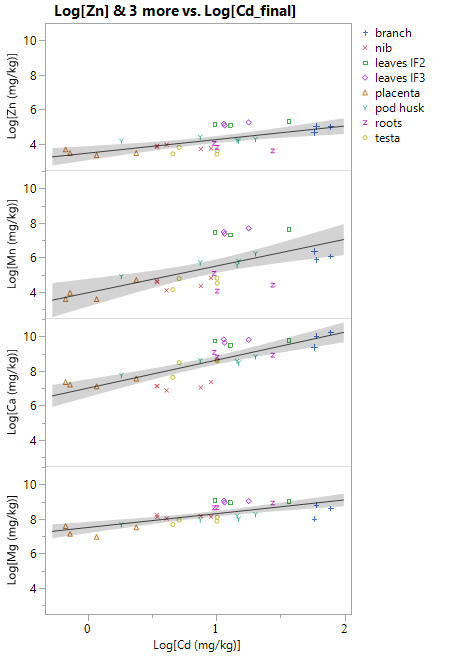

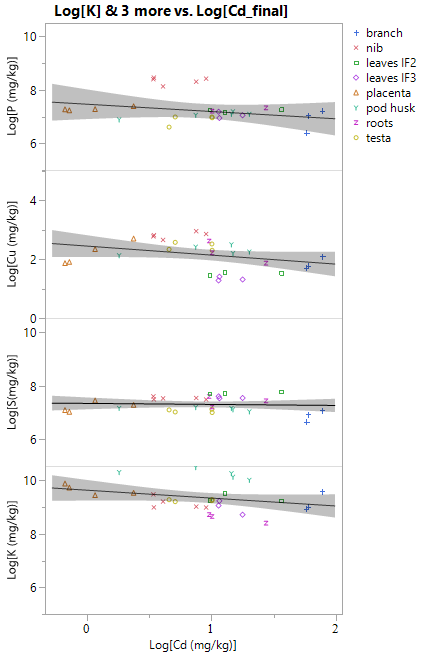


**Figure S5:** Relationships between the log transform of Cd concentrations and nutrients in the various organs, all values measured are shown for the n=3 trees. The grey zone is the confidence region for the linear fit (α = 0.05, Table 4). Left: Nutrients in group I correlate positively with Cd, Right: Group II elements correlate negatively with Cd in the various tissues.


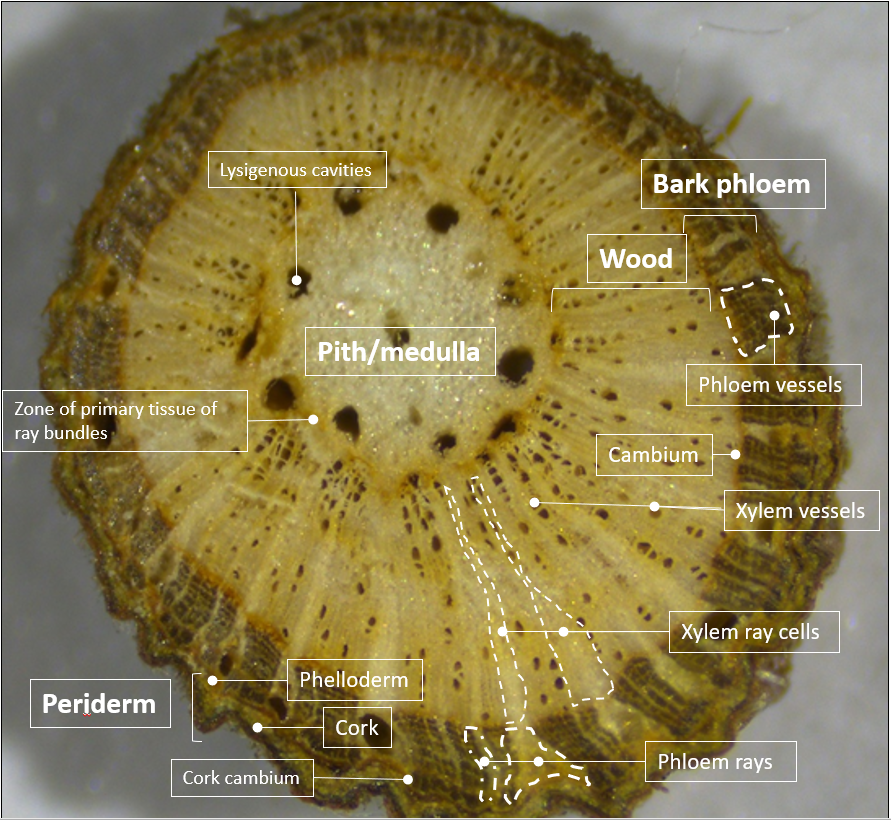


**Figure S6.** Anatomy of transverse cross section of cacao branch (NA T2). The pith in the center is surrounded by the wood, which is enclosed by the bark phloem and the cork.


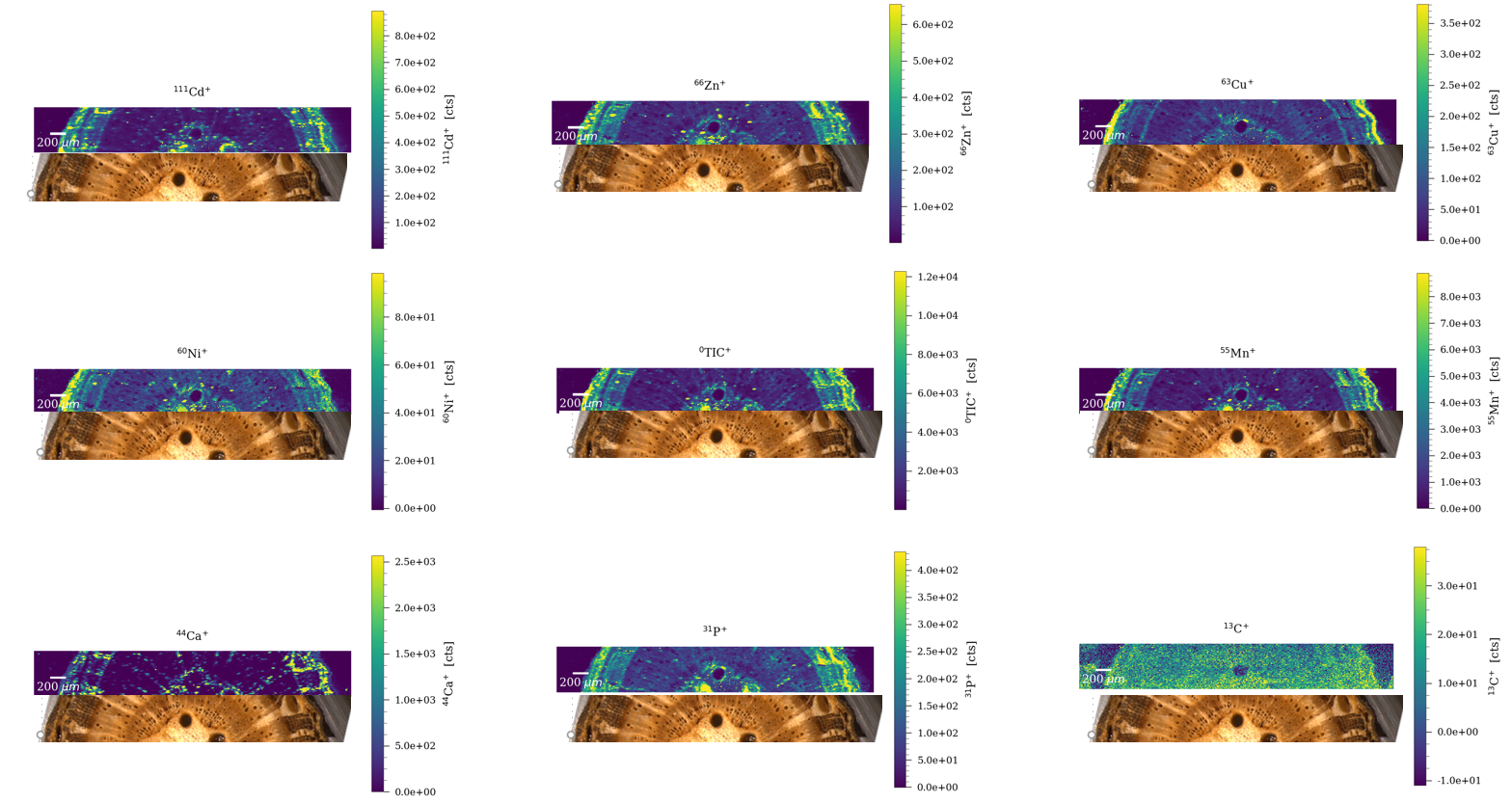


**Figure S7.** Microscope image and LA-ICP-MS elemental maps of cross section of branch NA T4. The intensity is expressed in total counts (cps). The color scale is adjusted to the range of each element. The trends were the same as observed in NA T2. Cd, Ca, Mn and Zn co-localized, and less spatial correlation was found between Cd and P or Cu.

# References

Argüello, D., Chavez, E., Lauryssen, F., Vanderschueren, R., Smolders, E. and Montalvo, D. (2019) ‘Soil properties and agronomic factors affecting cadmium concentrations in cacao beans: A nationwide survey in Ecuador’, *Science of The Total Environment*, 649, pp. 120–127. Available at: https://doi.org/10.1016/j.scitotenv.2018.08.292.

Bataille, C., Baldacchino, G., Cosson, R.P., Coppo, M., Trehen, C., Vigneron, G., *et al.* (2005) ‘Effect of pressure on pulse radiolysis reduction of proteins’, *Biochimica et Biophysica Acta - General Subjects*, 1724(3), pp. 432–439. Available at: https://doi.org/10.1016/j.bbagen.2005.04.021.

Huguet, S., Bert, V., Laboudigue, A., Barthès, V., Isaure, M.P., Llorens, I., *et al.* (2012) ‘Cd speciation and localization in the hyperaccumulator Arabidopsis halleri’, *Environmental and Experimental Botany*, 82, pp. 54–65. Available at: https://doi.org/10.1016/j.envexpbot.2012.03.011.

Huguet, S., Isaure, M.P., Bert, V., Laboudigue, A., Proux, O., Flank, A.M., *et al.* (2015) ‘Fate of cadmium in the rhizosphere of Arabidopsis halleri grown in a contaminated dredged sediment’, *Science of the Total Environment*, 536, pp. 468–480. Available at: https://doi.org/10.1016/j.scitotenv.2015.07.026.

Lewis, C., Lennon, A.M., Eudoxie, G. and Umaharan, P. (2018) ‘Genetic variation in bioaccumulation and partitioning of cadmium in Theobroma cacao L.’, *Science of the Total Environment*, 640–641, pp. 696–703. Available at: https://doi.org/10.1016/j.scitotenv.2018.05.365.

McBride, M.B., Frenchmeyer, M., Kelch, S.E. and Aristilde, L. (2017) ‘Solubility, structure, and morphology in the co-precipitation of cadmium and zinc with calcium-oxalate’, *Journal of Colloid and Interface Science*, 486, pp. 309–315. Available at: https://doi.org/10.1016/j.jcis.2016.09.079.

McKie, V.A. and McCleary, B. V. (2016) ‘A novel and rapid colorimetric method for measuring total phosphorus and phytic acid in foods and animal feeds’, *Journal of AOAC International*, 99(3), pp. 738–743. Available at: https://doi.org/10.5740/jaoacint.16-0029.

Murphy, J. and Riley, J.P. (1962) ‘A modified single solution method for the determination of phosphate in natural waters’, *Analytica Chimica Acta*, 27(1), pp. 31–36. Available at: https://doi.org/10.18393/ejss.477560.

Nurafiza, A., Tee, Y.., Boney, M., Albert, L.., Rozita, O. and Isa, I. (2017) ‘Carbon, Nitrogen, Phosphorus, and Potassium Content Partitioning of Cocoa Tree Parts in Serian, Sarawak’, *Pelita Perkebunan (a Coffee and Cocoa Research Journal)*, 33(3), p. 180. Available at: https://doi.org/10.22302/iccri.jur.pelitaperkebunan.v33i3.292.

Vanderschueren, R., Argüello, D., Blommaert, H., Montalvo, D., Barraza, F., Maurice, L., *et al.* (2021) ‘Mitigating the level of cadmium in cacao products: Reviewing the transfer of cadmium from soil to chocolate bar’, *Science of the Total Environment*, 781, p. 146779. Available at: https://doi.org/10.1016/j.scitotenv.2021.146779.
